# A gibberellin-assisted study of the transcriptional and hormonal changes occurring at floral transition in peach buds (*Prunus persica* L. Batsch)

**DOI:** 10.1186/s12870-024-05360-6

**Published:** 2024-07-08

**Authors:** Francesco Girardi, Monica Canton, Francesca Populin, Verónica Tijero, Giorgia Bettio, Sergi Munné-Bosch, Angela Rasori, Valerio Cardillo, Guglielmo Costa, Alessandro Botton

**Affiliations:** 1 Department of Agronomy, Food, Natural resources, Animals and Environment - DAFNAE, University of Padova, Agripolis, Viale dell’università 16, Legnaro, PD 35020 Italy; 2 Department of Evolutionary Biology, Ecology and Environmental Sciences, University of Barcelona, Diagonal 643, Barcelona, 08017 Spain; 3 Department of Agricultural and Food Sciences – DISTAL, University of Bologna, Bologna, 40126 Italy; 4 Present Address: Berry Genetics and Breeding Unit - Research and Innovation Centre (CRI), Fondazione Edmund Mach, San Michele all’Adige, Trento, 38098 Italy; 5 Present Address: Fruit Production Programme, Institute of Agrifood Research and Technology (IRTA), Parc Agrobiotech Lleida, Parc de Gardeny, Edifici Fruitcentre, Lleida, 25003 Spain

**Keywords:** Thinning, Floral transition, Peach buds, Gene expression, Gibberellins

## Abstract

**Background:**

Flower load in peach is an important determinant of final fruit quality and is subjected to cost-effective agronomical practices, such as the thinning, to finely balance the sink-source relationships within the tree and drive the optimal amount of assimilates to the fruits. Floral transition in peach buds occurs as a result of the integration of specific environmental signals, such as light and temperature, into the endogenous pathways that induce the meristem to pass from vegetative to reproductive growth. The cross talk and integration of the different players, such as the genes and the hormones, are still partially unknown. In the present research, transcriptomics and hormone profiling were applied on bud samples at different developmental stages. A gibberellin treatment was used as a tool to identify the different phases of floral transition and characterize the bud sensitivity to gibberellins in terms of inhibition of floral transition.

**Results:**

Treatments with gibberellins showed different efficacies and pointed out a timeframe of maximum inhibition of floral transition in peach buds. Contextually, *APETALA1* gene expression was shown to be a reliable marker of gibberellin efficacy in controlling this process. RNA-Seq transcriptomic analyses allowed to identify specific genes dealing with ROS, cell cycle, T6P, floral induction control and other processes, which are correlated with the bud sensitivity to gibberellins and possibly involved in bud development during its transition to the reproductive stage. Transcriptomic data integrated with the quantification of the main bioactive hormones in the bud allowed to identify the main hormonal regulators of floral transition in peach, with a pivotal role played by endogenous gibberellins and cytokinins.

**Conclusions:**

The peach bud undergoes different levels of receptivity to gibberellin inhibition. The stage with maximum responsiveness corresponded to a transcriptional and hormonal crossroad, involving both flowering inhibitors and inductors. Endogenous gibberellin levels increased only at the latest developmental stage, when floral transition was already partially achieved, and the bud was less sensitive to exogenous treatments. A physiological model summarizes the main findings and suggests new research ideas to improve our knowledge about floral transition in peach.

**Supplementary Information:**

The online version contains supplementary material available at 10.1186/s12870-024-05360-6.

## Background

Flowering is generally divided into two major steps: (i) the initiation of flower primordia (i.e., floral initiation) as a results of floral induction occurring mainly in leaves, which causes the transition of the meristems from vegetative to reproductive state, and (ii) the differentiation and development of mature flowers that undergo anthesis [1]. Floral initiation occurs with no visible external indications and includes all the developmental stages necessary for the irreversible commitment by the meristem to produce flowers [2]. Its control is not restricted to the developing meristem but may involve signals coming from other organs and from the external environment, such as ambient temperature [3, 4] and photoperiodic signals [5].

After floral induction initiates, the process may be quantitatively affected by correlative inhibitions and stress factors [6]. Floral induction occurs when an inductive environmental stimulus (i.e., photoperiod, low/high temperatures, etc.) is perceived and transduced into signals by endogenous integrators, which finally lead the meristem to pass from the vegetative to the reproductive phase, in which a series of changes take place in bud tissues [7]. Endogenous and environmental cues are often relayed and modulated by different hormones, thus conferring further developmental flexibility to the floral process under varying conditions [8]. The relative importance and the type of interactions between the regulatory factors and floral induction may substantially differ among the species, pointing out discrepancies especially between annual and perennial plants [9].

In *Prunus* species, different genes and proteins complexes have been linked to the regulation of flowering [10–13], even if this process is not completely understood. Canonical floral initiation genes, such as *FLOWERING LOCUS T* (*FT*), *CONSTANS* (*CO*), *SUPPRESSOR OF OVEREXPRESSION OF CONSTANS 1* (*SOC1*), and *TERMINAL FLOWER 1* (*TFL1*), have been identified and partially characterized in fruit trees [14–19].

Phytohormones, such as gibberellins (GAs) and cytokinins (CKs), may significantly affect floral transition in fruit trees, with the former generally showing an inhibitory role, in contrast to the commonly described stimulatory action played in annual species [20–22]. In apple and cherry, exogenous GA applications prevented floral initiation, while GA inhibitors and high CKs/GAs ratios promoted it [21–23]. Since the 60s, it was demonstrated that applications of gibberellin A3 (GA_3_) from full bloom up to early summer can reduce the number of flower buds and fruit load in *Prunus* species [22, 24–29].

In peach orchards, thinning is indispensable and usually performed by hand. However, hand thinning is an expensive, labour-intensive practice, and the skilled workforce needed to perform this operation is increasingly difficult to find [30, 31]. The purpose of flower/fruitlet thinning in fruit trees is dual: (i) to reduce fruit load, improve sink-source balance and decrease competition among fruits, thus obtaining bigger fruits with improved fruit-quality parameters at harvest, and (ii) to avoid biennial bearing by removing the source of floral induction inhibition (i.e., the fruits), especially in terms of inhibitory signals, such as the GAs [32, 33].

A very detailed review recently published by Costa and Botton [34] showed an exhaustive view of thinning in peach. In this crop, fruit thinning is generally less effective than flower thinning, especially for early ripening cultivars in which the fruit developmental cycle is shorter and thus the time to take advantage of the reduction of fruit load is not sufficient to achieve the same results as with early flower removal. Therefore, the best solution in peach would be to start from an optimal number of flowers, which allows to obtain high-quality fruits without inhibiting floral transition. Besides supporting breeding programs aimed at selecting new “self-thinning” genotypes, such as for apple [35], it is fundamental also in peach to understand the factors controlling flower induction, initiation, and differentiation.

In order to shed light on how bud sensitivity to endogenous signals changes during its transition to the reproductive phase, i.e. when morphological changes associated with the floral state are not visible yet, the physiological background of untreated buds was characterized by means of a transcriptomic time-course analysis accompanied by hormone profiling. Moreover, a GA_4/7_-based product was concurrently applied at the same timepoints to inhibit floral transition and identify the most sensitive stages. A physiological model was proposed to describe these important bud developmental phases as related to the return bloom measured in the following season.

## Methods

### Trial design and sample collection

The main field trial has been carried out in 2019 on the flat-nectarine cultivar Platinet 1 (*Prunus persica* var. *platycarpa*). This cultivar is harvested approximately 22 days before the reference cultivar Redhaven. The experiment was conducted at Vivai Zanzi’s farm in Ferrara, Italy (44°46’56.355”, 11°39’55.419”). An appropriate number of homogeneous trees with a suitable fruit load, all protected by anti-hail nets, were selected and marked among 40 trees in total to build a single block experiment with five trees per block. Blocks, including an untreated control (UTC), were separated by two untreated trees to avoid drift effects. The commercial formulation Sevengib (10 g/L GA_4/7_ with a prevalent content of GA_7_; Fine Agrochemicals Ltd, Whittington, Worcester, UK) was sprayed at 100 mg/L by using a shoulder sprayer until run-off, at 45 (T1), 51 (T2), 58 (T3), 65 (T4), and 79 (T5) days after full bloom (DAFB). Treatments timing was decided according to available preliminary data provided by the manufacturer of Sevengib, indicating the maximum sensitivity to the treatment at around 60 DAFB. Consistently, the experiment time-course was centered at 58 DAFB and implemented with two additional timepoints before (45 and 51 FAFB) and after (65 and 79 DAFB) the optimal application time. Before each spraying (Additional File 1), a suitable number of untreated shoots were collected in three biological replicates (about 10 shoots, 5 from each side of the row, per replicate, sampled from the intermediate portion of the canopy well-exposed to light) and the axillary buds from the apical and basal halves of the shoot excised separately (about 40–50 buds per type for each replicate), frozen in liquid nitrogen and stored at -80 °C for following RNA-Seq analyses and qPCR validation. Buds were sampled in the same way also at 115 DAFB, in this case from both GA-treated and UTC trees, to be used for qPCR to measure *APETALA1* gene expression, as described below. During winter, the trees were pruned according to standard practices.

### Return bloom measurements

In the following year (spring 2020), return bloom, i.e. the amount of functional flowers when the tree returns to bloom, was assessed in all the experimental blocks by means of photographic measurements carried out at full bloom. Pictures of each tree of all the blocks were taken from both sides of the row using a dark background and elaborated using ImageJ software to isolate the total area of the picture covered by the flowers and then compare the mean value of the five trees with the mean of UTC, resulting into a percentage with respect to the latter.

### RNA isolation, RNA-Seq analyses and qPCR

Total RNA was extracted from buds using Spectrum™ Plant Total RNA Kit (Sigma-Aldrich, St. Louis, MO, USA) following the manufacturer’s instructions. RNA concentration and quality was determined by measuring OD_260/230_ and OD_260/280_ ratios by means of a NanoDrop 2000c spectrophotometer (Thermo Scientific, Waltham, MA, USA).

RNA of untreated bud samples collected at T1, T3 and T5 was used for RNA-Seq analyses using an Illumina NextSeq 500 platform (Illumina, San Diego, CA, United States). These samples were chosen according to the efficacy of the treatments in terms of return bloom: T3 showed the maximum efficacy, while T1 and T5 were those more significantly different from T3. The whole RNA-Seq procedure was carried out following manufacturer’s instructions.

qPCR was carried out as described by Botton et al. [36] on both untreated and treated buds collected at 115 DAFB. Both the genes *TEF2* (*PRUPE_4G138900*) and *UBQ10* (*PRUPE_4G204900*) were used for normalization [37]. All the primers used in qPCR are listed in Table 1.

**Table 1:** Sequences of the primers used for qPCR gene expression analyses

Gene ID	Gene Name and abbreviation	Primer Name	Sequence (5’-3’)
*PRUPE_1G290600*	*APETALA1 (AP1)*	AP1_Fw	AGACCGCTCTTAAACAAATTCGATCA
		AP1_Rev	CCTTCTCCTTGATCTTCTTTGCCAA
*PRUPE_6G128400*	*CENTRORADIALIS-like (CEN)*	CEN_Fw	GTTATGACCGATCCAGATGTTCCCG
		CEN_Rev	GTGTTATCAGTTGTGCCTGGGA
*PRUPE_6G021200*	*Cyclin B1.2 (CYCB1.2)*	CYCB1.2_Fw	GAAGGCGGCAGTGGTAGTAG
		CYCB1.2_Rev	TAACTCCACCAGCCATAGCC
*PRUPE_4G269600*	*Cyclin B1.4 (CYCB1.4)*	CYCB1.4_Fw	GCTGGGATGGTGCCTAACAGT
		CYCB1.4_Rev	AGCCGCAGAATGAAAAGTGTGG
*PRUPE_6G364900*	*FLOWERING LOCUS T (FT)*	FT_Fw	CCCGCTTGTTGTTGGAAGAGTG
		FT_Rev	AGTCCTAAGATCATCCCCACCAG
*PRUPE_4G150200*	*Gibberellin 2-oxidase 6 (GAox6)*	GA2ox6_Fw	GGTTTTGACCAATGGGAGGTTCCA
		GA2ox6_Rev	GATTGCAGTGGAGCTATTTTCTCAC
*PRUPE_3G161500*	*Gibberellin-regulated family protein RSI-1 (RSI-1)*	RSI-1_Fw1	CTGCTTCTTCTACTCACATTCTCTGATG
		RSI-1_Rev1	ACGCCTGGAGGAACACATAGG
*PRUPE_4G204900*	*Polyubiquitin 10 (UBQ10)*	UBQ10_Fw	AAGGCTAAGATCCAAGACAAAGAG
		UBQ10_Rev	CCACGAAGACGAAGCACTAAG
*PRUPE_6G256300*	*Squamosa Promoter binding protein-like 9.1 (SPL9.1)*	SPL9.1_Fw	GGGTGTGAATTTGGCTCAGTC
		SPL9.1_Rev	CAAGACCAGCAACAATGACCATG
*PRUPE_7G074200*	*Squamosa Promoter binding protein-like 9.2 (SPL9.2)*	SPL9.2_Fw	GACCACGAGAACTGAGCGAGTA
		SPL9.2_Rev	CCAGGTATTCAAGTAGGCATTCATC
*PRUPE_7G112600*	*TERMINAL FLOWER1 (TFL1)*	TFL1_Fw	TGTTTCACCCCAACAACAAAAATGT
		TFL1_Rev	AAGGAGGGTTCACAGACTGC
*PRUPE_4G138900*	*Translation Elongation Factor 2 (TEF2)*	TEF2_Fw	GGTGTGACGATGAAGAGTGATG
		TEF2_Rev	TGAAGGAGAGGGAAGGTGAAAG
*PRUPE_5G176400*	*Trehalose Phosphate Synthase 9 (TPS9)*	TPS9_Fw	GCCTCTGATTCCCCTCGTACAAGT
		TPS9_Rev	GGACCAGTAAGCCACATCATGAG
*PRUPE_1G256200*	*Trehalose Phosphate Synthase 10 (TPS10)*	TPS10_Fw	AAGAAATTGTGGAGCCTGTGATGA
		TPS10_Rev	CAACCAATCCTTTGCTTACTCCC

### Bioinformatic analyses

Raw FASTQ files were uploaded to the Gene Expression Omnibus (GEO) database (https://www.ncbi.nlm.nih.gov/geo/) with the accession number GSE247681. RNA-seq reads were quality-checked using FastQC (Brabaham Bioinformatics, Cambridge, United Kingdom). All the reads were mapped onto the newest version of the peach *P. persica* genome v.2.0.a1 [38]. The softwares Hisat2 [39] and Samtools [40] were adopted to index the genome, align the reads, and manage the different file formats, while HTSeq [41] was used to generate the counts according to gene models available in public GFF3 files. Read counts of each sample were merged into a count matrix for the following analyses. Differential gene expression analyses were performed with the Bioconductor package DESeq2 [42], while functional analyses were performed with a double approach, either on DEGs through enrichment analyses or using a wider procedure of pathway analysis with PGSEA algorithm [43]. To this aim, the iDEP bioinformatic platform v1.1 was used for a more integrated approach to data analysis [44].

Correlative network analysis was carried out with R software (https://www.r-project.org) through the package igraph v1.5.1 [45] based on Pearson’s pairwise correlations. Only genes (nodes) linked to hormones by correlations (edges) higher than 0.90 were kept in the graph. Prim’s algorithm [46] was used to convert the graph adjacency object into a minimum spanning tree. The network graph with a default layout based on the force-directed algorithm by Fruchterman and Reingold [47] was exported from R to the software Cytoscape v3.10.1 [48] for visualization, by using the package RCy3 v2.23.0 [49]. Within Cytoscape, nodes were colored according to their eccentricity with their size reflecting the mean expression level (only for genes), while edges’ color indicates the type of correlation (direct or inverse). Few aesthetic modifications (i.e. transparency of nodes and edges, bending of the edges connecting the subnetworks) were made manually for an optimized visualization.

### Hormone analyses

For phytohormones analyses, which was carried out only on untreated T1, T3 and T5 basal buds, 100 mg of lyophilized bud tissue sample was extracted as previously described in Botton et al. [36]. Abscisic acid (ABA), the auxin indole-3-acetic acid (IAA), jasmonic acid (JA) and its conjugate jasmonoyl-isoleucine (JA-Ile), salicylic acid (SA), the cytokinins *trans*-zeatin (tZ) and 2-isopentenyl adenine (2-iP), gibberellins A3, A4 and A7 (GA_3_, GA_4_ and GA_7_) were analyzed by UHPLC-ESI-MS/MS using the MRM (multiple reaction monitoring) mode for quantification as previously described [50]. Quantification was made by calculating recovery rates for each sample by using internal standards of deuterium-labeled compounds (OlChemIm Ltd., Olomouc, Czech Republic).

### Statistical analyses

Multiple comparison statistics were calculated within R Studio (Version 2023.06.0 + 421) with R version 4.2.3 as described by Botton et al. [36].

## Results

### *APETALA1* is a reliable quantitative indicator of flower initiation in peach buds

The efficacy of the different treatments was tested on both basal and apical buds sampled from all the experimental blocks (compared to the untreated control, UTC) at 115 DAFB, when floral initiation is supposed to be mostly accomplished according to microscopic analyses [51]. To this aim, the expression of *APETALA1* (*PpAP1*) gene (*PRUPE_1G290600*) was chosen as known marker of reproductive identity of the meristems [52–54], to assess if there is also a quantitative relationship between its expression levels and the return bloom assessed in the following spring.

**Fig. 1:**
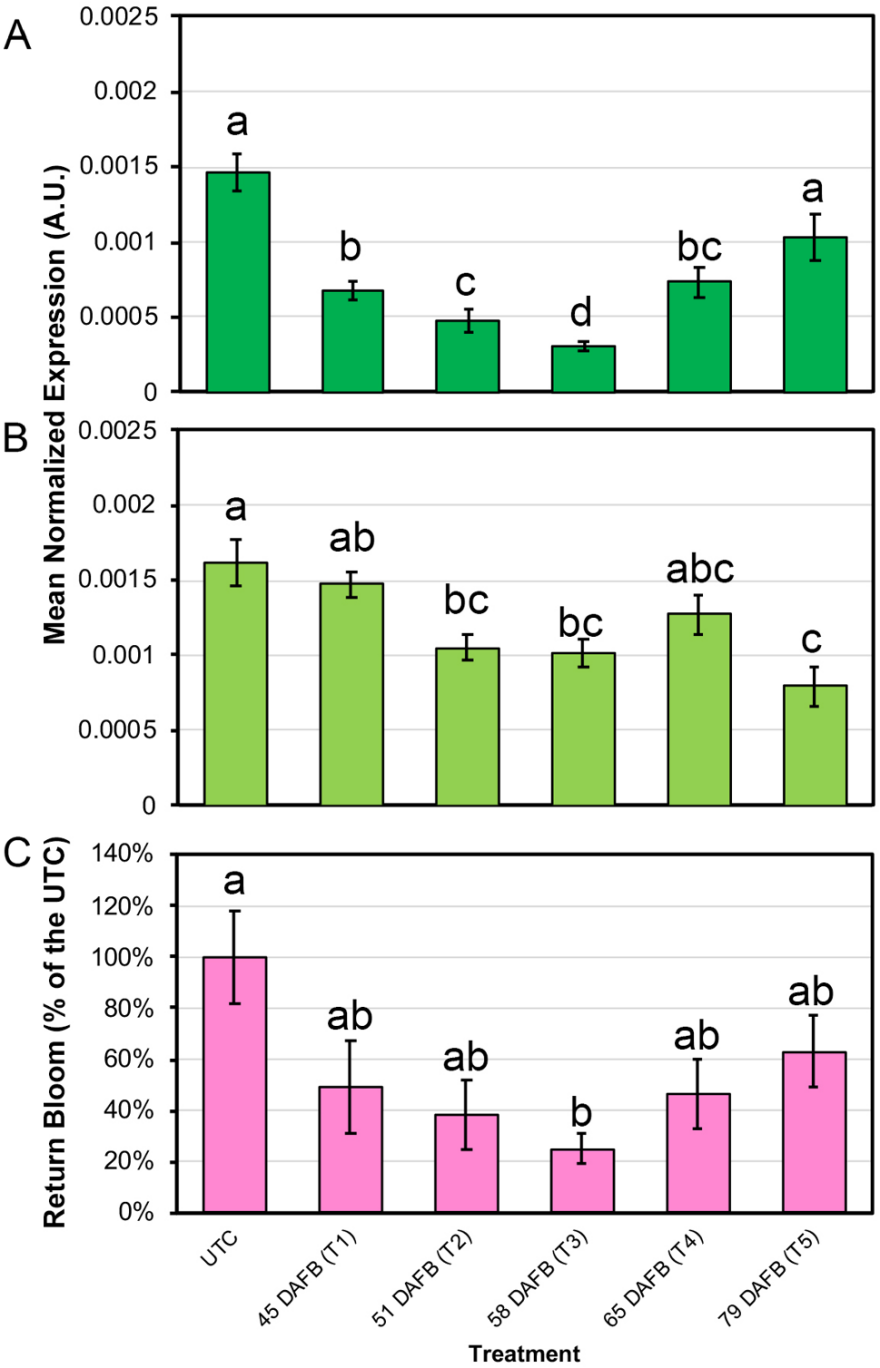
*APETALA1* expression in buds and return bloom measurements. qPCR expression of *APETALA1* in basal (**A**) and apical (**B**) buds collected from untreated peach trees (UTC) and from trees treated with GA_7_ at 45, 51, 58, 65, and 79 Days After Full Bloom (DAFB). Letters indicate statistically significant differences (*P* < 0.05; *n* = 6) and bars show standard error. A.U., Arbitrary Units. (**C**) Return bloom reported as a percentage of the UTC. Letters indicate statistically significant differences (*P* < 0.05; *n* = 5) and bars show standard deviation

In basal buds, *PpAP1* expression levels decreased from T1 to T3 and then increased again until T5 (Fig. 1A). Its maximum levels of transcripts were measured in the UTC buds. In apical buds, the expression pattern was almost similar to the basal ones, except for a markedly low transcript amount measured at T5 (Fig. 1B).

Return to bloom assessed in both treated and untreated trees at anthesis displayed a very clear situation (Additional File 2). A “V-shaped” gradient was shown by treated trees with a minimum at T3. Moreover, all the treated trees showed a lower number of flowers with respect to the UTC (Fig. 1C), indicating that the treatment was effective at all times of application.

Therefore, a clear correlation (Pearson’s equal to 0.99 on average) was found between the expression of *PpAP1* in basal buds and return to bloom, given by the same buds that were not removed by pruning during winter.

### RNA-Seq profiling in peach buds during development

The transcriptional changes occurring in untreated peach buds in the different developmental stages (the same as treatments’ application) were analyzed using an RNA-Seq approach, aimed at shedding light on the physiological determinants of the differential efficacy of GA_7_ in inhibiting floral transition, as resulted from return bloom assessments. At this step, both the basal and apical buds were analyzed, and summary statistics are reported in Table 2. On average, the number of RNA-Seq reads obtained was equal to 37.7 million, with a mean overall alignment rate equal to 97.5%.

**Table 2:** Summary of RNA-Seq statistics in all replicates of apical and basal buds

	**Apical buds**								
	**T1**			**T2**			**T3**		
	**Rep1**	**Rep2**	**Rep3**	**Rep1**	**Rep2**	**Rep3**	**Rep1**	**Rep2**	**Rep3**
Total No. Reads	34,292,026	34,434,870	43,732,266	44,203,838	23,233,490	43,383,946	46,708,006	42,615,230	30,979,324
Reads not aligned concordantly	2,280,843	2,342,888	2,513,036	2,530,210	1,580,338	2,858,636	3,599,642	3,399,374	2,337,941
Reads aligned concordantly 1 time	30,781,724	30,935,613	39,616,754	39,185,197	20,645,436	38,090,729	40,907,378	37,334,992	26,745,504
Reads aligned concordantly > 1 time	1,229,459	1,156,369	1,602,476	2,488,431	1,007,716	2,434,581	2,200,986	1,880,864	1,895,879
Overall alignment rate	98.00%	98.11%	98.11%	98.25%	97.39%	97.69%	97.73%	98.09%	97.79%
	**Basal buds**								
Total No. Reads	28,158,584	36,578,606	32,471,348	33,367,686	38,858,384	48,217,022	54,349,109	29,073,897	34,389,536
Reads not aligned concordantly	1,808,904	3,306,449	2,403,535	2,936902	3,594,670	6,108,572	4,261,492	2,122,271	2,638,820
Reads aligned concordantly 1 time	25,386,919	32,042,393	28,758,558	28,953,456	33,552,465	40,111,704	46,158,581	25,317,958	29,830,188
Reads aligned concordantly > 1 time	962,761	1,229,764	1,309,255	1,477,328	1,711,249	1,996,746	3,929,036	1,633,668	1,920,528
Overall alignment rate	98.06%	97.59%	98.01%	96.78%	96.88%	96.57%	96.99%	96.74%	97.00%

The heatmap obtained with the 2,000 most variable genes showed three main expression patterns, which were clustered together (Additional File 3). Cluster A included the apical buds at T1 and T3, with one replicate of the latter behaving like an outlier. Cluster B comprised the basal buds at T1 and T3, with the three biological replicates clustering together. Finally, cluster C grouped together all the T5 samples, showing again the replicates of each type of buds forming separate subclusters.

**Fig. 2:**
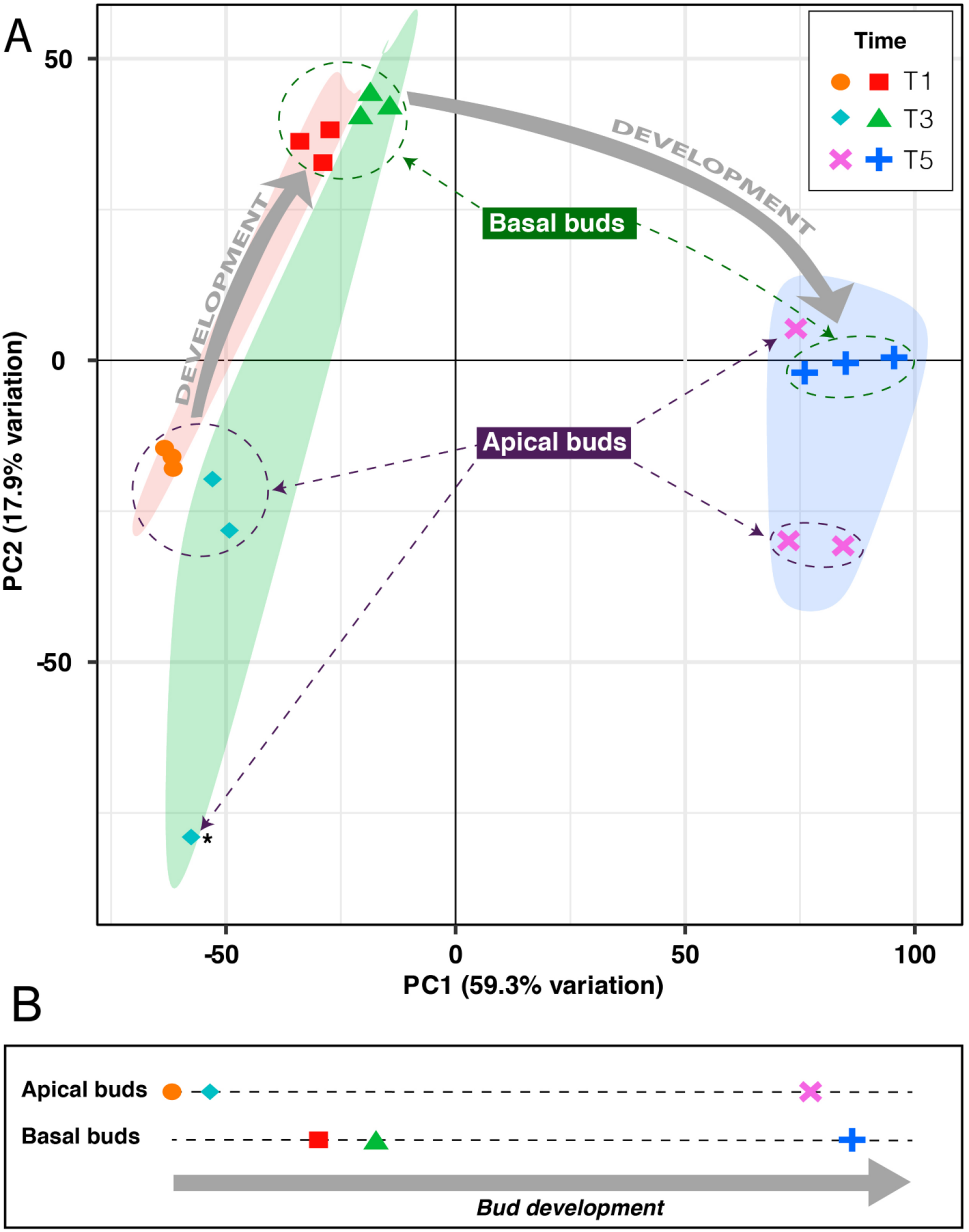
PCA of RNA-Seq data and developmental timing of apical and basal buds. (**A**) T1, T3 and T5 samples are shown in the PCA with red, green and blue shadings, respectively. The variation explained by the first two PCs is shown in the axes. The asterisk marks an outlier of apical buds, while the grey arrows indicate a likely developmental line followed by the buds’ transcriptomes. (**B**) Positions of the different sampling timepoints of both apical and basal buds with respect to development according to the first PC

The two-dimensional Principal Component Analysis (PCA) of the transcriptomic data (Fig. 2A) revealed that the first two principal components (PCs) explain most of the variance (77.2%), and samples from each time point of each type of bud clustered together, indicating the high quality of the biological replicates, except for the outlier, as already pointed out in the heatmap. PC1 was correlated with time (*P* = 1.16e-08), while PC2 was correlated with bud position (*P* = 1.70e-03). In detail, T1 and T3 buds’ transcriptomes were located very close one another, but apical and basal buds were quite distant in the PCA, with the former in the bottom-left and the latter in the top-left quadrant. At T5, the transcriptomes were closer, although still separated, except for one single replicate of apical buds, which clustered adjacent to the basal ones. PCA not only substantially confirmed the heatmap results, but also allowed to trace a hypothetical developmental time-course comprising the different types of buds. Assuming that PC1, being correlated with time, may represent the variation of the buds’ transcriptomes, their one-dimensional projection showed that the apical buds at T1 and T3 are at a similar developmental stage. The basal buds at T1 are in a more advanced stage, followed very close by T3 buds. Apical buds seem to recover their delay at T5, reaching a developmental phase comparable to basal ones (Fig. 2B).

### Identification of DEGs related to treatment efficacy

For DEGs identification, only the transcriptomes of basal buds were used, as most of the apical ones are usually removed through winter pruning and, consequently, lie outside the practical focus of this study. Using DESeq2 with an FDR cutoff of 0.01 and a twofold change cut-off in expression level, the DEGs were identified using the efficacy of the treatment as a factor (model = expression ~ efficacy). Two levels of efficacy were linked to the samples, either low, for T1 and T5, or high, for the T3 sample with the lowest return bloom (Fig. 1C).

**Fig. 3:**
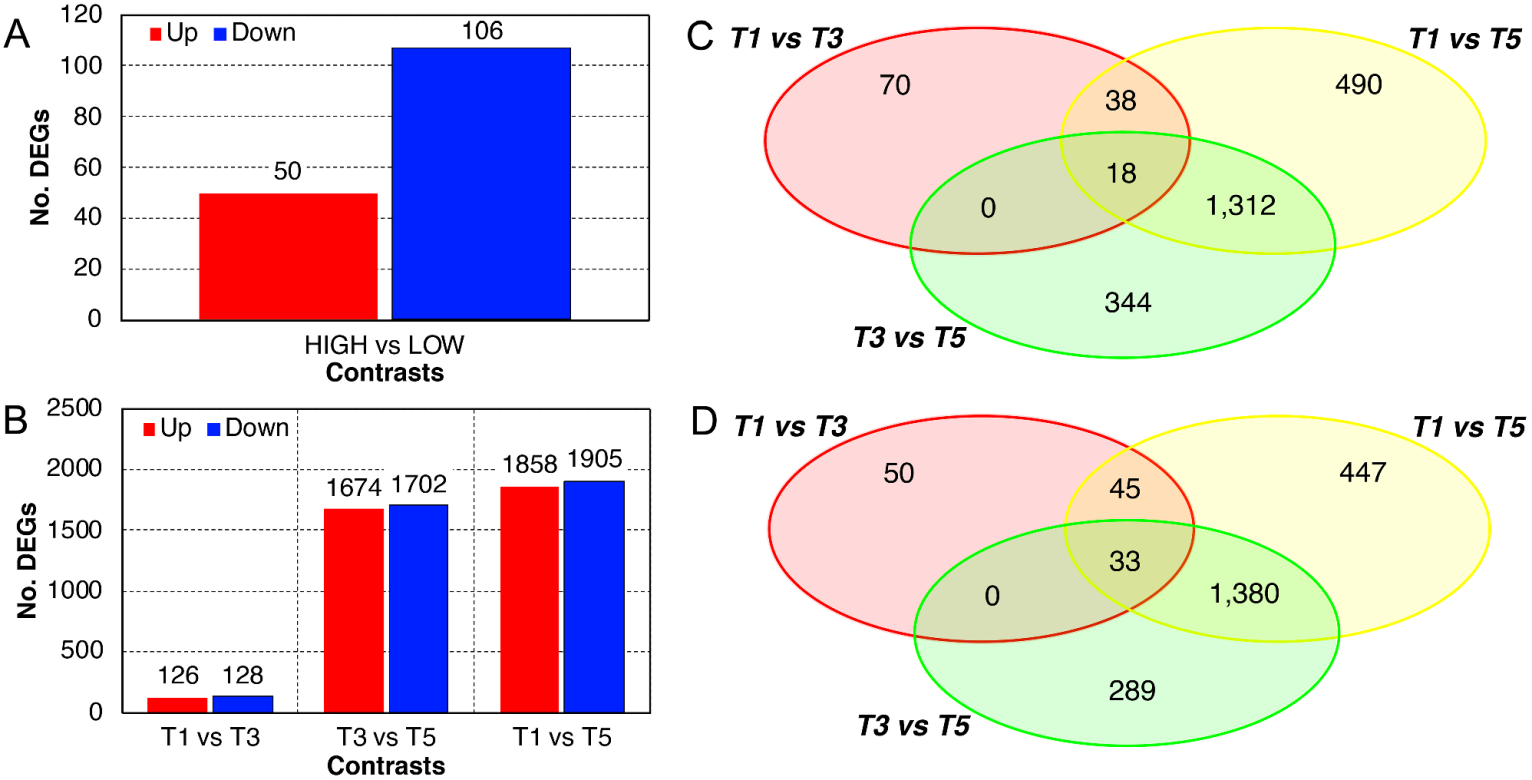
Differentially Expressed Genes (DEGs) and Venn diagrams. Bar charts show the number of DEGs in the contrast between samples according to the efficacy of the treatment (**A**) or the timepoints (**B**). Up- and down-regulated genes are shown in red and blue, respectively. Venn diagrams show up- (**C**) and down-regulated (**D**) genes in the different contrasts

A total of 156 DEGs were identified, among which 50 and 106 were up- and down-regulated, respectively, when treatment’s efficacy was higher (Fig. 3A). Enrichment analysis using the Gene Ontology (GO) biological processes sub-vocabulary gave significant results only for the downregulated genes, with the terms “*response to reactive oxygen species*”, “*response to hydrogen peroxide*”, “*protein complex oligomerization*”, “*response to temperature stimulus*”, and “*response to heat*” being over-represented in this subset of genes (Additional File 4). The complete list of DEGs can be found in Additional File 8.

### Identification of DEGs related to bud development

Considering time as a factor (expression ~ time; T1 vs. T3, T1 vs. T5 and T3 vs. T5) with the same statistical parameters used above, the number of DEGs was generally higher than with the previous approach and was fully consistent with clustering analyses (Fig. 3B). In the contrast T1 vs. T3, the number of DEGs was the lowest, being equal to 254 (126 up- and 128 down-regulated in T1 with respect to T3). The different transcriptional backgrounds shown by T3 and T5 samples in both the heatmap and the PCA turned into 1,674 and 1,702 up- and down-regulated genes, respectively, in T3 with respect to T5. Finally, the contrast between the two extremes, T1 and T5, displayed the highest number of DEGs: 3,763, including 1,858 and 1,905 up- and down-regulated genes, respectively. Only 18 and 33 genes are up- and down-regulated, respectively, in both the first two contrasts, while most of the genes that are DEGs from T3 to T5 are differentially expressed also from T1 to T5 (Fig. 3C-D). The functional analysis of the DEGs identified in the first contrast (T1 vs. T3) pointed out enriched GO terms dealing with, among the most interesting, “*detection of oxygen*”, “*response to abiotic stimulus*”, and “*response to auxin*”, only in the up-regulated genes (Additional File 5). The other important contrast (T3 vs. T5) showed enriched GO terms in both up- and down-regulated genes. The former group of DEGs was enriched by functions regarding “*polysaccharide metabolic process*”, “*cell wall organization or biogenesis*”, and “*photosynthesis*”, while the down-regulated genes were functionally enriched by terms dealing with “*response to chemical*”, “*transcription DNA-templated*”, and “*fruit ripening climacteric*” (Additional File 6). The complete list of DEGs can be found in Additional File 8.

### Pathway enrichment analysis

**Fig. 4:**
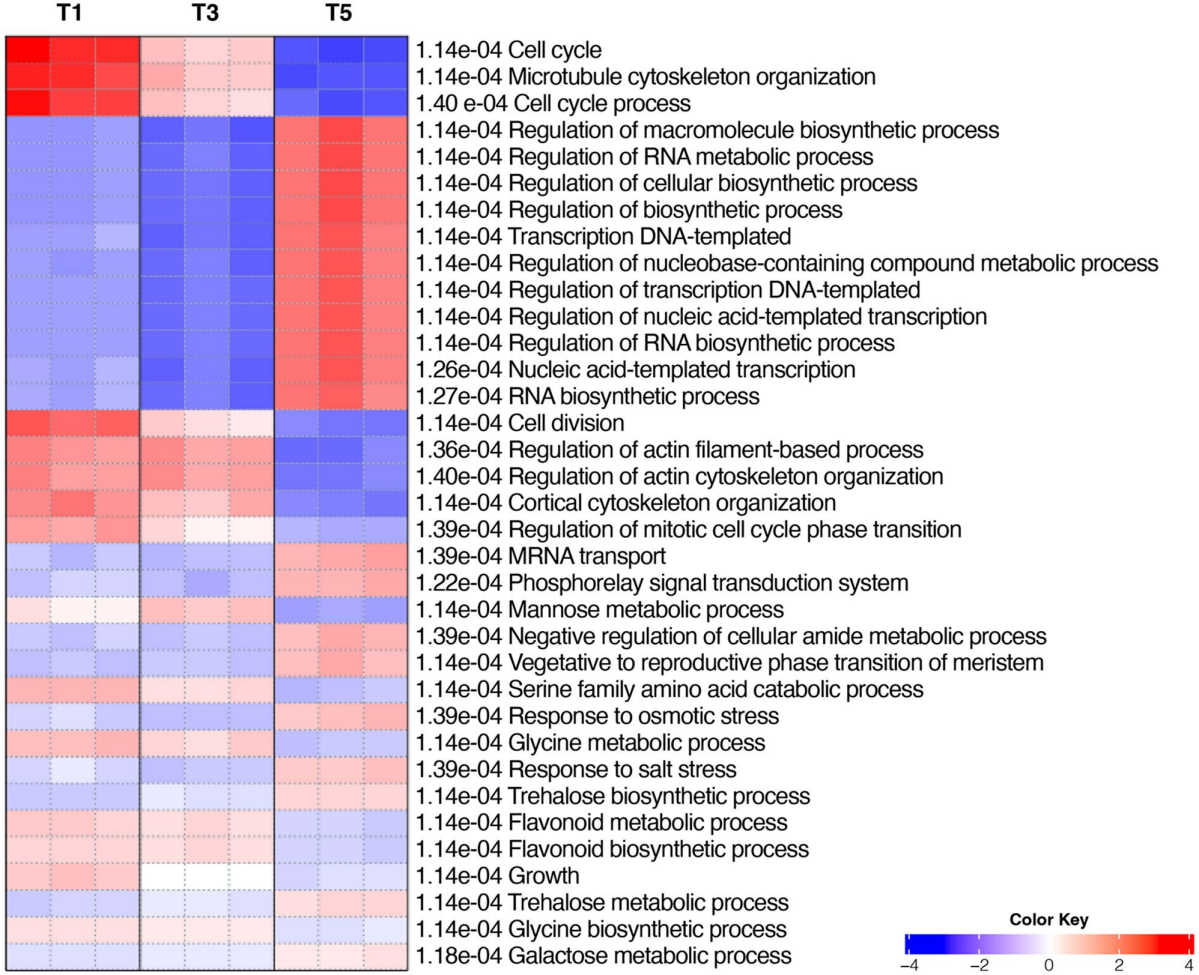
PGSEA analysis heatmap with all samples. The FDR value is shown for each GO biological process terms representing the enriched pathways. A color scale indicates the degree of up- and down-regulation of the whole pathway

In order to improve the functional information, an additional approach was adopted with a pathway analysis, using the PGSEA method with all samples [43]. This method uses all the expression data of genes that can be mapped to a certain pathway with Gene Ontology or other annotation sets (in this case the GO biological process sub-vocabulary was used), and a statistical analysis is carried out taking the pathway as a whole, to assess if it is significantly affected or not in the different samples. In other words, this method tests if the different pathways display an expression trend supported by statistical significance (pathway significance cut-off FDR = 0.05). The annotation tree showing the hierarchy of the GO terms (Additional File 7) may help in identifying the most relevant pathways shown in the PGSEA heatmap (Fig. 4), such as “*Cell cycle*”, which is progressively down-regulated from T1 to T5, “*Vegetative to reproductive phase transition of meristems*” and “*Trehalose biosynthetic process*”, both up-regulated from T3 to T5, and “*Flavonoid biosynthetic process*”, another interesting pathway that is down-regulated from T3 to T5. All these processes may be potentially involved in bud development and/or floral induction [55–58].

### Real-time quantitative PCR

A subset of genes was chosen either among the DEGs, based upon the functional analyses, or directly from pathways that are considered relevant for this study, regardless of their statistical significance, in order to validate the RNA-Seq results through quantitative real-time PCR (qPCR), including also the apical buds. Gene annotations were retrieved from GDR database (www.rosaceae.org) based upon *Prunus persica genome v2.0.a1*, while gene names were decided upon the available annotations (including blast matches with Arabidopsis) to provide the best information about gene function.

**Fig. 5:**
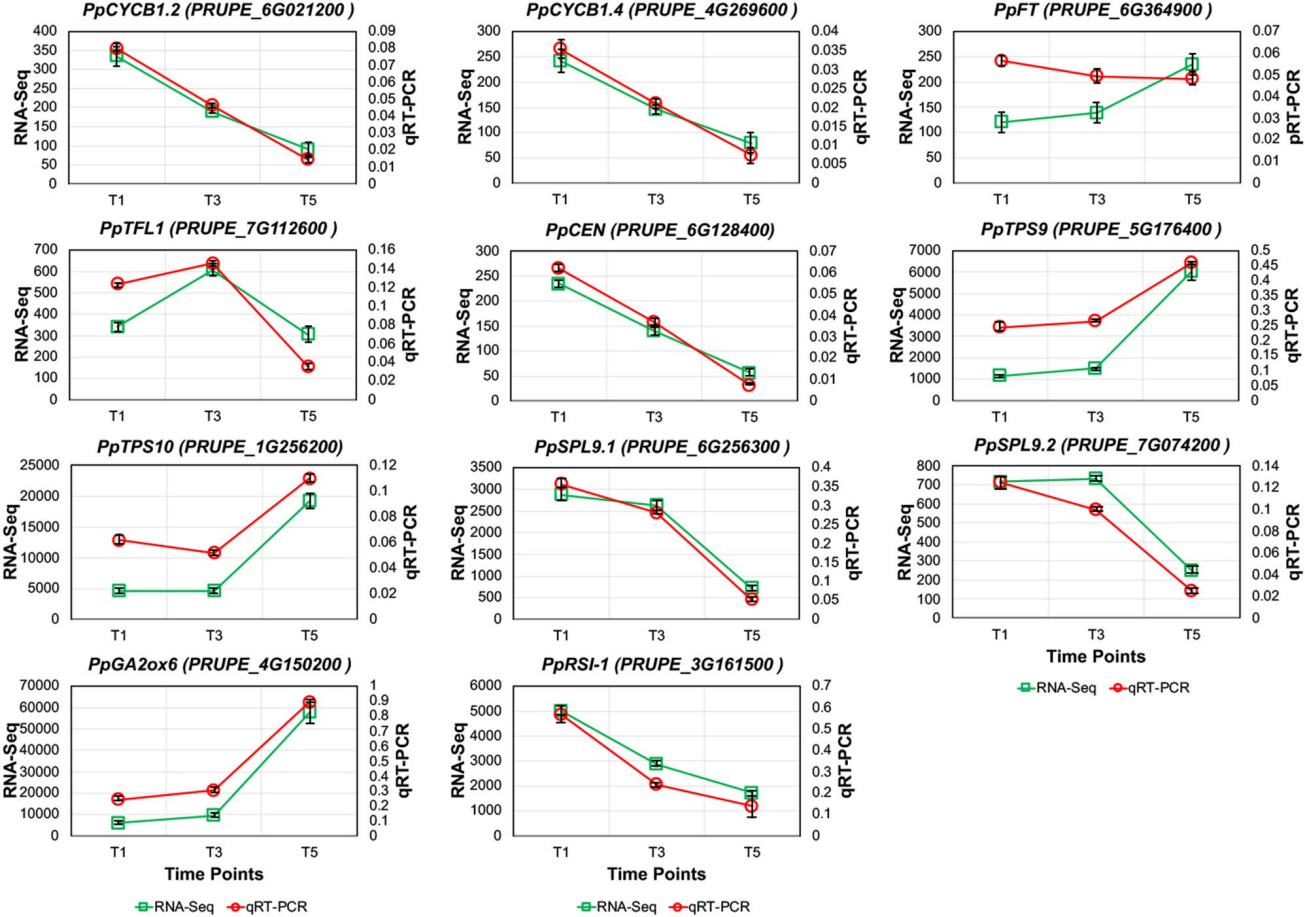
qPCR validation of RNA-Seq in basal buds. Expression values of eleven genes are reported for both the qPCR (red lines; as arbitrary units of mean normalized expression) and RNA-Seq (green lines; as RPKM, reads per kilobase of transcript per million reads mapped). Gene name and ID are indicated at the top of each chart. Bars, where visible, indicate standard error

Concerning the “*Cell cycle*” function, two cyclin B1-encoding genes were selected, namely *PpCYCB1.2* (*PRUPE_6G021200*) and *PpCYCB1.4* (*PRUPE_4G269600*). B1-type cyclins control microtubule organization during cell division [59]. Their expression patterns fully matched the RNA-Seq results, with Pearson’s correlation coefficients as high as 0.99 and 0.94 and levels of transcripts progressively decreasing through bud development (Fig. 5).

The most important pathway pointed out by PGSEA analysis, i.e. “*Vegetative to reproductive phase transition of meristems*”, included a relevant number of genes, from which three were chosen for the validation: *PpFT* (*PRUPE_6G364900*; [60]), *PpTFL1* (*PRUPE_7G112600*; [61]), and *PpCEN* (*PRUPE_6G128400*; [62]). While *PpTFL1* and *PpCEN* showed a very strong correlation between qPCR and RNA-Seq results (0.80 and 0.94, respectively), *PpFT* was validated with a Pearson’s coefficient equal to 0.62, due to a low correlation in basal buds. Among all the genes validated through qPCR, *PpFT* showed the lowest levels of correlation, and its pattern of expression was substantially stable in basal buds and increasing from T1 to T5 in apical buds. In both types of buds, *PpTFL1* showed its highest expression at T3, dropping thereafter at T5, while *PpCEN* transcripts decreased through the experiment (Fig. 5).

For the “*Trehalose biosynthetic process*” pathway, the genes selected were both encoding Trehalose Phosphate Synthases (TPS), i.e.,* PpTPS9* (*PRUPE_5G176400*) and *PpTPS10* (*PRUPE_1G256200*). Their expression in both basal and apical buds was stable from T1 to T3 and increased at T5, showing a very good correlation with RNA-Seq results (0.95 and 0.97, respectively).

Concerning the genes chosen among the DEGs, *PRUPE_6G256300* (*PpSPL9.1*) and *PRUPE_7G074200* (*PpSPL9.2*), coding for two SQUAMOSA Promoter-Binding Protein-Like (SPL) transcription factors, were shown to be stably expressed at T1 and T3 and then significantly down-regulated from T3 to T5, concurrently showing a strong correlation equal to 0.97 and 0.95, respectively, with expression values measured through RNA-Seq (Fig. 5). Considering the importance of gibberellin inhibition of floral transition in trees [20–22] and, specifically, in stone fruit species [63], gibberellin-related genes were searched among the DEGs, resulting in two genes, i.e. *PRUPE_4G150200*, coding for the Gibberellin 2-oxidase PpGA2ox6 known to be induced by gibberellins [64], and *PRUPE_3G161500*, encoding a gibberellin-regulated GASA/GAST (Gibberellic Acid Stimulated Arabidopsis/Gibberellin Stimulated Transcript) protein [65], similar to RSI-1 of tomato and PmGASA15 of *Prunus mume* [66], the latter shown to be rapidly induced in leaves by gibberellin treatments performed in a fruit crop of the same genus as peach. *PpGA2ox6* expression was stable from T1 to T3 and then significantly increased at T5, while *PpRSI-1* was progressively decreasing from T1 to T5 (Fig. 5). Both genes showed a strict correlation between qPCR and RNA-Seq data, with Pearson’s coefficient equal to 0.99 and 0.96, respectively.

### Hormone profiling

**Fig. 6:**
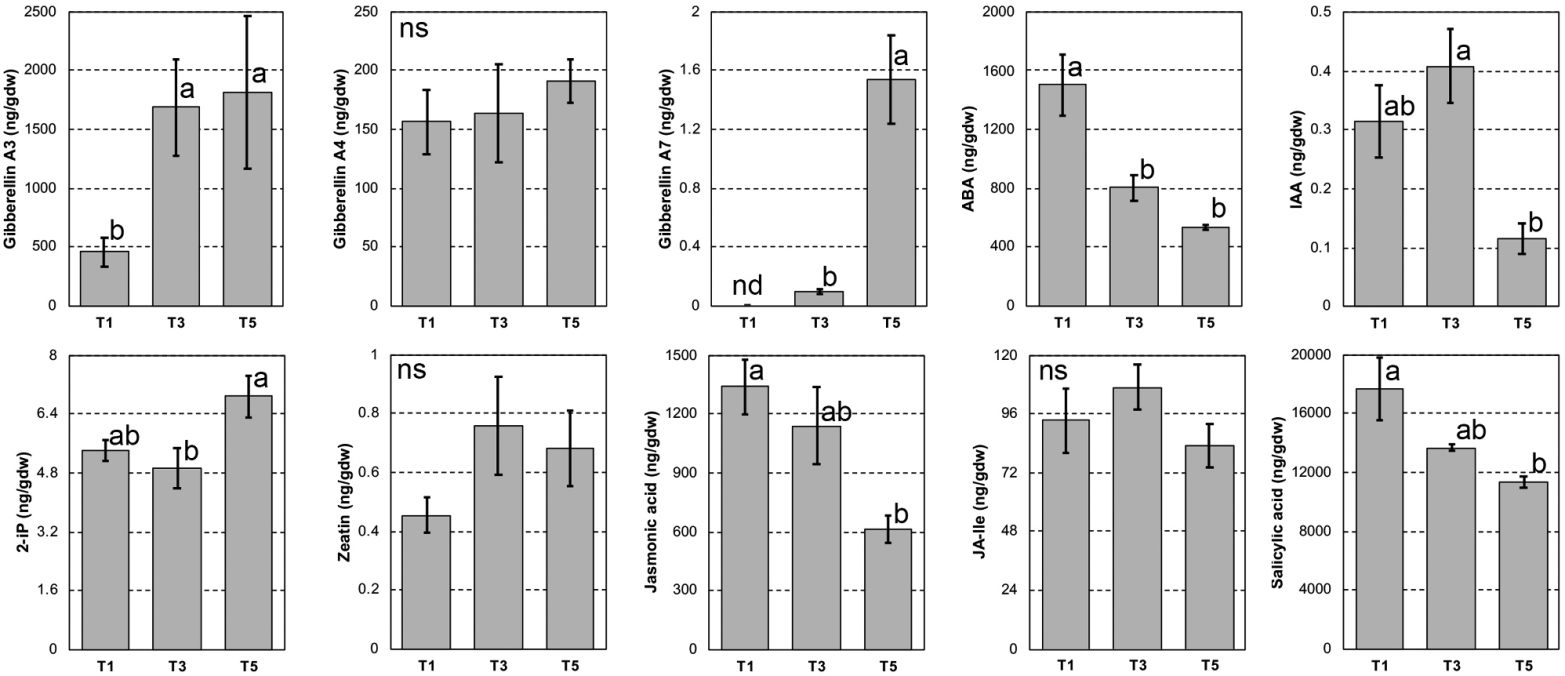
Hormone levels measured at T1, T3 and T5. The names of the hormones are shown on the left side of each chart. Letters indicate statistically significant differences (*P* < 0.05; *n* = 5) and bars show standard error

Hormone profiling is shown in Fig. 6, while all the gene expression analyses regarding the biosynthetic and signal transduction pathways of each hormone are displayed in the Supplementary Material.

GAs showed a general increasing trend, with GA_3_ increasing from T1 to T3, GA_7_ reaching its maximum at T5, and GA_4_ not varying significantly. This general increase of gibberellins was accompanied by a strong transcriptional activation of their biosynthetic genes from T3 to T5, preceded by a weak activation of their signal transduction pathway at T3, visible as an increased expression of *DELLA* genes (Additional File 9), the latter being previously known to correlate with GAs levels [67].

ABA showed decreasing levels throughout the experiment, in spite of the upregulation of both its biosynthetic and signal transduction elements from T3 to T5 (Additional File 10). IAA levels pointed out a similar trend, even though the expression of both signal transduction and biosynthetic genes did not show any correlation (Additional File 11).

tZ did not change significantly, while 2-iP increased at T5. In this case, the data pointed out by both the biosynthetic genes and the signal transduction elements were fully consistent. Indeed, the genes encoding isopentenyltransferases (IPT), which catalyze a rate-limiting step of cytokinin biosynthesis [68], were upregulated from T3 to T5, concomitantly with the downregulation of tZ biosynthetic branch and with the strong upregulation of Type-A ARR, known to be positively regulated by cytokinins [69] (Additional File 12).

SA and JA showed closely similar decreasing patterns from T1 to T5, while JA-Ile levels did not differ significantly. Both the jasmonate biosynthetic and signal transduction pathways did not show consistent results (Additional File 13). In case of SA, however, the PR-1 genes, notoriously induced by this hormone in Arabidopsis [70], were strongly repressed throughout the whole experiment (Additional File 14).

Finally, although ethylene was not quantified, both its rate-limiting step of biosynthesis (i.e. the genes coding for ACC synthase) and signal transduction pathway especially concerning the Ethylene Responsive Factors (ERFs) genes from T3 to T5, were upregulated (Additional File 15).

### Hormones-genes correlative network

**Fig. 7:**
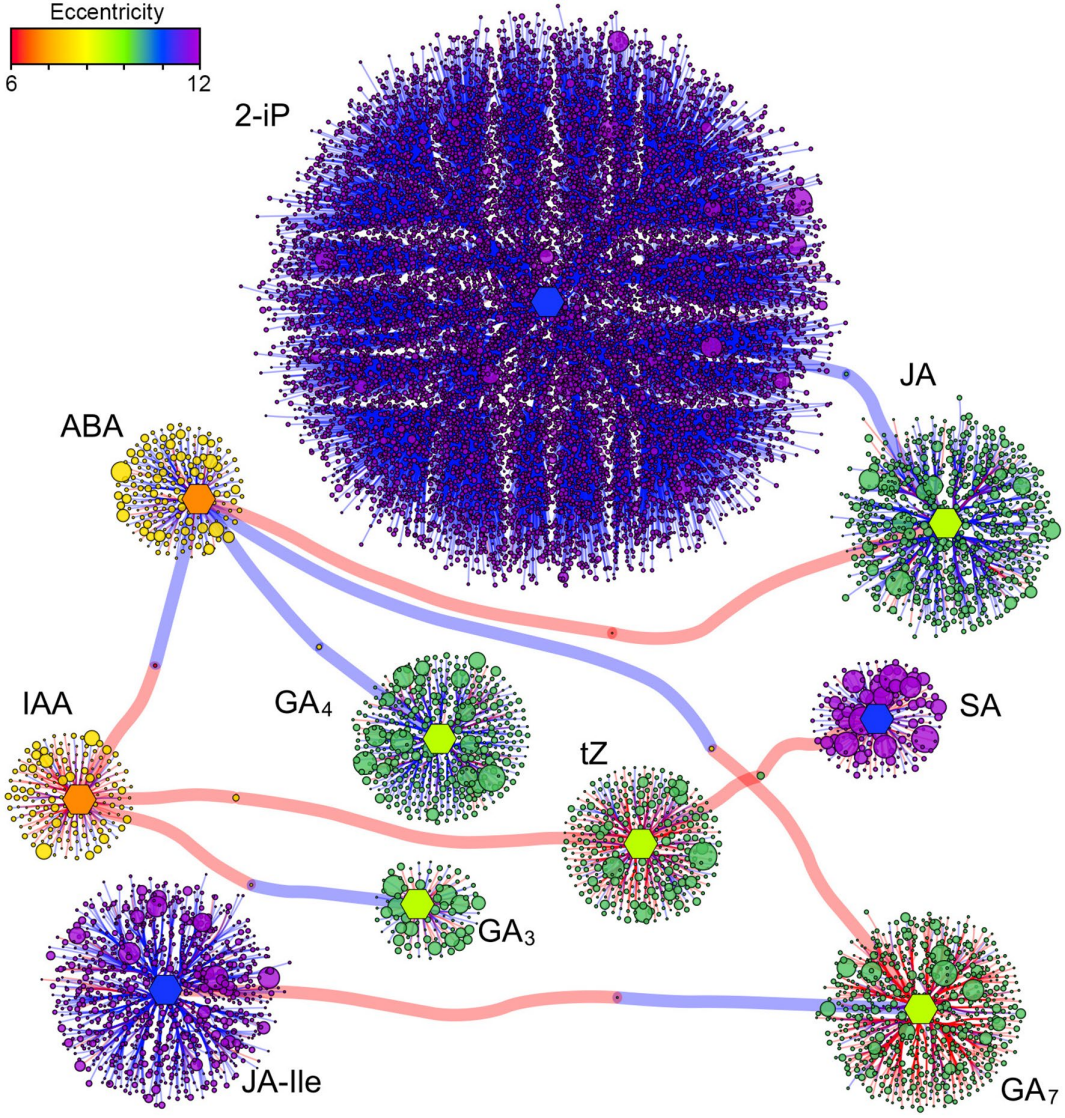
Correlative network of hormones and genes. Direct and inverse correlations higher than 0.90 are shown with red and blue edges, respectively. Hormones are represented as hexagonal nodes of identical size. Genes are shown with circular nodes, whose size reflects the mean expression level and color is based upon the level of eccentricity, as shown by the top-left scale. The layout of the network is based on the force-directed algorithm by Fruchterman and Reingold (1991). 2-iP, 2-isopentenyladenine; ABA, abscisic acid; GA_3/4/7_, gibberellin A3, A4 and A7; IAA, indoleacetic acid; JA, jasmonic acid; JA-Ile, jasmonoyl-isoleucine; SA, salicylic acid; tZ, trans-zeatin

Assuming that a high correlation between the level of expression of a certain gene and the amount of a specific hormone may indicate a functional relationship between them (i.e., the hormone may regulate the expression of that gene), a correlative network was built including hormones and genes linked by a Pearson’s correlation index higher than 0.90, including both direct and inverse correlations, with the aim to identify the main gene functions controlled by the hormones (Fig. 7).

The network comprises a total of 18,146 nodes including also the 10 bioactive hormones analyzed above. Most of the genes (14,385) were correlated with the levels of the cytokinin 2-iP, either positively (166) or negatively (14,219). Enrichment analysis of gene functions pointed out a long list of significant (FDR < 0.05) GO terms, among which “*cellular response to stress*”, “*cellular macromolecule biosynthetic process*”, “*RNA metabolic process*” and “*gene expression*” are the most representative. A complete list of enriched GO terms can be found in Tables S3-S7. All the other subnetworks grouped with a lower number of genes, starting from the JA-Ile network, comprising a total of 905 nodes, of which 780 and 124 inversely and directly correlated to the hormone levels, respectively. The most significant enriched GO terms were “*cytoskeleton organization*”, “*transmembrane transport*”, “*inorganic anion transport*”, “*purine ribonucleoside triphosphate catabolic process*” and “*intracellular protein transport*”. The JA subnetwork composed by 784 nodes was kept together by 670 and 113 negative and positive correlations, respectively, with no significantly enriched GO terms. The following network was the GA_7_, with 668 nodes connected by 545 and 122 positive and negative relationships, respectively. In this case, the most specific overrepresented gene functions were “*photosynthesis*,* light harvesting*” and “*generation of precursor metabolites and energy*”. GA_4_ subnetwork, composed by 446 nodes, mostly linked to this gibberellin by negative correlations (357) and only few (88) by positive indexes. The genes of this subnetwork were enriched by functions dealing with “*cellular macromolecule biosynthetic process*”, in particular “*1*,*3-beta-glucan biosynthetic process*”. The tZ subnetwork included 345 nodes, linked by 229 and 115 direct and inverse correlations, and was enriched by functions such as “*cellular macromolecule biosynthetic process*” and “*translation*”. ABA network was composed by 194 nodes, kept together by 45 and 148 positive and negative correlations, respectively. No significant enrichment was pointed out, such as for the SA subnetwork, made by 168 nodes with 63 and 104 direct and inverse correlations, respectively. The network of the most important plant hormone, IAA, was built with 167 nodes, linked by 115 and 51 positive and negative relationships, respectively, with no significant enriched function. Finally, the GA_3_ subnetwork had 92 nodes, kept together by 46 and 45 positive and negative correlations, respectively. No enrichment was found.

On the overall correlative network, also the synergisms and antagonisms between and among hormones can be deducted from the type of correlation that links the different subnetworks through the bridging nodes (Fig. 7). Therefore, synergisms may exist between 2-iP and JA, and among IAA, tZ and SA, while antagonisms may occur between ABA and GA_7_, ABA and IAA, IAA and GA_3_, GA_7_ and JA-Ile.

## Discussion

All the transcriptomic studies available in recent literature regarding peach bud development were carried out mostly during dormancy [71–76]. Transcriptomics was recently applied to study flowering induction and floral bud differentiation in sweet cherry [77], although this research was mostly focused on the latter developmental stages, when flower structures differentiate. Moreover, several targeted gene expression studies have been carried out in the past that point out the possible functions of some canonical floral initiation genes, as already discussed above and reviewed by Penso et al. [78]. However, a complete physiological overview of the transcriptional changes occurring in the most crucial stages of peach bud floral transition is still missing, and such a knowledge gap becomes even harder considering the importance of this information as related to practical issues, such as thinning and fruit quality. For this reason, we tried to take advantage of a new GA_4/7_-based formulation for reducing return bloom, to focus on those crucial stages of floral transition when bud’s destiny is decided.

Gene expression studies of *PpAP1* and return bloom pointed out a clear relationship between time of application and final efficacy of the GA_4/7_ treatment (Fig. 1). These statistically supported results indicate also that the expression of *PpAP1* measured in peach buds at middle/late summer is a good quantitative predictor of return bloom in this kind of thinning approaches, where UTC trees are available as a reference, and it can be eventually used in the future for this purpose.

The RNA-Seq analyses proved to be effective in discriminating the different developmental stages of the collected samples, in both basal and apical buds (Fig. 2). The position-dependent developmental kinetics followed in the first two timepoints indicated that from T1 to T3 the buds may have started to collect and integrate the environmental signals that initiate flowering, regardless from their position, and then made the “great developmental jump” to T5. Considering only the basal buds and based on the efficacy of GA_4/7_ treatments, the physiological stages represented in the present experiment can be described as follows: T1, developing bud with low sensitivity to flowering signals; T2, developing bud with increasing sensitivity to flowering signals; T3, competent bud with maximum sensitivity to flowering signals; T4, transiting bud with decreasing sensitivity to flowering signals, partially induced to flower; T5, transiting bud with low sensitivity to flowering signals, almost completely induced to flower transition. When the bud starts to become sensitive to environmental signals and the optimal conditions occur, floral transition is a very fast process, so that the time passed from T1 to T3 (two weeks) and from T3 to T5 (three weeks) may not be proportional to the “developmental distance” travelled by the bud, which is much bigger from T3 to T5. For this reason, the T5 samples appeared very different from the previous ones, at least from a transcriptional point of view, as testified also by the number of DEGs found in the different contrasts (Fig. 3).

The analysis of DEGs dealing using the *ex post* treatment efficacy as a factor pointed out that in the T3 buds, where GA_4/7_ was able to inhibit floral transition at the highest levels, processes dealing with response to ROS (Reactive Oxygen Species) and heat were downregulated. ROS were previously shown to have a pivotal role in the integration between temperature and light signals (reviewed by Krasensky-Wrzaczek and Kangasjärvi [79]) that may be involved in floral induction, and redox regulation is fundamental also in flowering [80]. Among the genes downregulated at T3, one encodes a thioredoxin (*PRUPE_1G410500*), which was also shown to interact with FT and positively regulate flowering in cotton [81]. Taking these indications into account, we can hypothesize that a ROS burst at T1/T2 may have facilitated the integration of environmental signals triggering floral evocation. Then ROS may have decreased at T3 and, when floral induction was partially achieved, started to increase again with active reproductive development at T4/T5.

The analysis of DEGs related to bud development pointed out that response to auxin is upregulated at T1 and this is consistent with previous results showing a positive role of this hormone in flowering promotion in several species, among which Arabidopsis [82, 83], strawberry [84], and saffron [85]. The contrast T3 vs. T5 revealed a generally increasing metabolic activity in terms of polysaccharide and cell wall organization, as well as an enhanced transcriptional activity, which are typical of an actively developing reproductive bud [86–88].

PGSEA analysis gave an extensive view of the pathways affected by floral transition and confirmed that sampling strategy was carried out correctly, by showing, among the other pathways, the upregulation of the “*Vegetative to reproductive phase transition of meristems*” (Fig. 4). This could not be taken for granted, due to the very small size of the buds sampled at early developmental stages with respect to the surrounding tissues, with the latter that could have biased the transcriptomic analyses.

The qPCR gene expression assays (Fig. 5), largely and significantly correlated with RNA-Seq, pointed out the role of specific genes during floral transition of peach buds. Two B-type cyclin genes showed a decreasing expression through the experiment, such as pointed out during flower initiation also in Arabidopsis meristems [89]. The expression of some canonical floral transition genes was fully consistent with their putative function, especially regarding *PpTFL1*, which showed an interesting peak of expression at T3. This gene is a known repressor of flowering in several species and horticultural crops [61, 90, 91] and seems to act in prolonging the vegetative phase, as demonstrated in grapevine [92]. Thus, its maximum expression at T3 may have enhanced the inhibitory effect of the GA_4/7_ treatment. Concerning another pathway emerged from the PGSEA analysis, i.e., trehalose biosynthesis, the expression of two TPS genes peaking at T5 was consistent with their essential function previously pointed out in floral evocation [55]. Mutants of these genes in Arabidopsis were shown to flower extremely late, even under otherwise inductive environmental conditions [57]. The downregulation of two *SPL* genes at T5 was again in line with previous findings, as these genes were shown to be positively involved in floral transition in several species and perennial crops, the latter including *Citrus* [32] and apple [93]. Finally, concerning the gibberellin-related genes, an important indication came out from expression analysis, especially regarding *PpGA2ox6*, indicating that gibberellin metabolism was deeply changing during the experiment. The latter indication was fully confirmed by the hormone profiling, showing a general increasing trend of GAs levels (Fig. 6), especially for GA_3_ and GA_7_ [22, 24–26, 28, 29, 94]. Besides the activation of GAs biosynthesis and signal transduction pathways shown through the KEGG maps (Additional File 9), the correlative network pointed out that GA_4_ and GA_7_ may control the expression of genes mainly dealing with “*cellular macromolecule biosynthetic process*” and “*generation of precursor metabolites and energy*”, respectively. This finding is in full agreement with data previously discussed and with literature [86–88], focusing on the activation of specific metabolisms once that the bud is transiting into the reproductive phase. We may hypothesize that anticipating this natural peak of GAs through GA_4/7_ application at T3 may have triggered this metabolic shift too early, when the bud was still in vegetative phase, thus stimulating the vegetative growth.

Concerning the other hormones, the most interesting findings regard the cytokinin 2-iP, which increased significantly at T5, oppositely to the expected antagonism with GAs, but consistently with the upregulation of both its biosynthesis and Type-A ARR genes as summarized by KEGG maps (Additional File 12). Indeed, the subnetwork of 2-iP was the biggest one, including 14,385 genes, almost all of them negatively correlated with the levels of the hormone. This may indicate that 2-iP positively regulates floral transition through the repression of genes involved in the vegetative growth, rather that only activating floral identity genes as shown before in Arabidopsis [95, 96], grapevine [97] and other species [98, 99]. The typical antagonism between GAs and ABA in controlling flowering time (as reviewed by Shu et al. [100]) was herein confirmed especially with respect to GA_4/7_, thus pointing out the pivotal positive role of this gibberellin among the others in controlling floral transition in peach. This antagonism was pointed out not only in terms of pattern of accumulation of both hormones (Fig. 6), but also by the correlative network with the putatively regulated genes (Fig. 7). IAA levels were also found to increase from T1 to T3 (Fig. 6). Auxin was shown to be essential for floral meristem initiation [101] and its increase at T3 further supports the idea that this is a critical step for floral transition in peach. A certain degree of antagonism emerged with respect to ABA and GA_3_, further supporting the positive role of auxin in reproductive phase transition of peach buds. Worthy to note also the different behaviors shown by JA and its conjugated form JA-Ile. While the former significantly decreased through the experiment, the latter displayed only non-significant oscillations and an antagonistic relationship with GA_4/7_ in terms of gene regulation. The role of jasmonates in flowering time regulation is still debated, although most of the available research often indicates a negative role of these hormones on this process (reviewed by Zhao et al. [102]). Therefore, based upon our results, a negative role in floral evocation may be hypothesized for JA also in peach.

## Conclusions

**Fig. 8:**
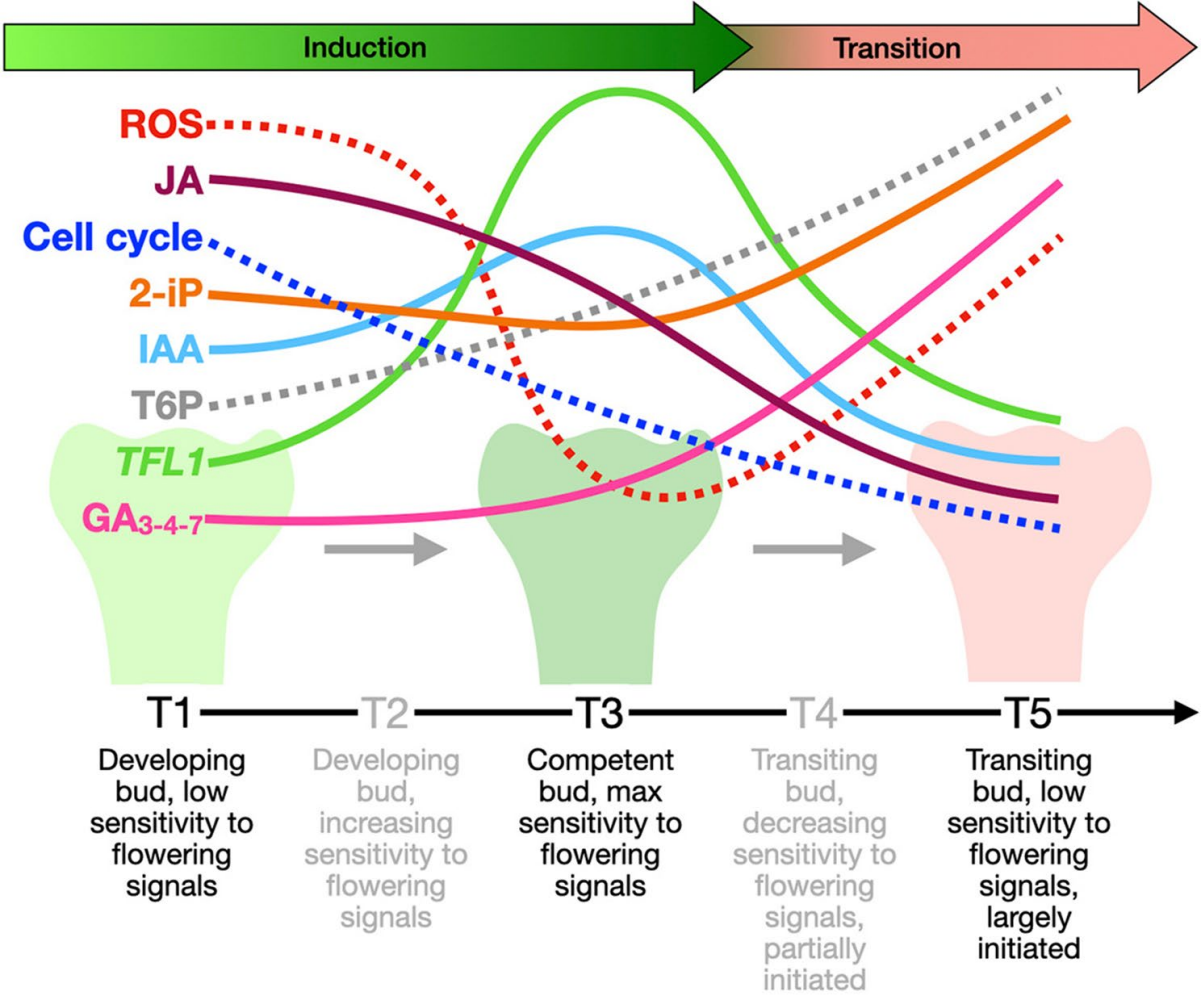
Physiological working model of peach bud development covering the floral initiation stages. The stages of bud development are described below each timepoint. Continuous lines indicate biological entities that were actually quantified, while dashed lines refer to compounds or processes whose levels or rates were inferred from gene expression data. ROS, reactive oxygen species; JA, jasmonic acid; 2-iP. 2-isopentenyladenine; IAA, indoleacetic acid; T6P, trehalose-6-phosphate; *TFL1*, *TERMINAL FLOWER 1*; GA_3 − 4−7_, gibberellin A3, A4 and A7

Taking all the results together, a working physiological model can be traced for floral transition in peach summarizing the involvement of the main regulatory factors at both genetic and hormonal level (Fig. 8). According to this model, bud’s acquisition of a full competence to respond to flowering signals may pass through a “sensitizing phase” with high ROS levels at T1, followed by their likely decrease at T3 and, possibly, a new increasing trend thereafter when bud reproductive development starts. Floral transition is also accompanied by a decrease of JA, a known inhibitor of the process, a reduced rate of cell division, an early and continuous increase of T6P, and a late increase of the cytokinin 2-iP, both the two latter players being positive regulators. IAA, whose role in floral initiation is still debated, peaks when the bud reaches its maximum sensitivity to flowering signals. At this developmental stage a still undifferentiated status is guaranteed by a peak of expression of *TFL*, a floral transition retardant, which may work as a marker of the most GA-sensitive developmental stage of the bud. After T3, when the bud is already initiated to the reproductive phase, gibberellins start to increase, thus closing the process with a saturation of the signal transduction pathway and a lower sensitivity to exogenous GA applications. Based upon this model, the specific treatment with GA_4/7_ applied at T3, may have anticipated the natural increase of GAs just when the bud is passing a sort of critical crossroad, when it is deciding its future destiny, thus achieving the highest inhibition of floral transition.

Besides putting together new information about gene expression and hormone levels during floral transition, these findings highlight the need for more comprehensive and targeted analyses to achieve a more detailed physiological view of the process in peach and, through comparative genomic approaches, in other tree crops.

### Electronic supplementary material

Below is the link to the electronic supplementary material.

Supplementary Material 1

Supplementary Material 2

Supplementary Material 3

Supplementary Material 4

Supplementary Material 5

Supplementary Material 6

Supplementary Material 7

Supplementary Material 8

Supplementary Material 9

Supplementary Material 10

Supplementary Material 11

Supplementary Material 12

Supplementary Material 13

Supplementary Material 14

Supplementary Material 15

Supplementary Material 16

## Data Availability

The datasets supporting the conclusions of this article are available in the Gene Expression Omnibus (GEO) repository, under the accession number GSE247681 (https://www.ncbi.nlm.nih.gov/geo/query/acc.cgi?acc=GSE247681).

## Abbreviations

ABA: Abscisic acid
CKs: Cytokinins
DAFB: Days after full bloom
DEGs: Differentially expressed genes
FDR: False Discovery Rate
GAs: Gibberellins
GO: Gene ontology
IAA: Indoleacetic acid
JA: Jasmonic acid
PCA: Principal component analysis
ROS: Reactive oxygen species
SA: Salicylic acid
T6P: Trehalose-6-phosphate
UTC: Untreated Control
