# Supplementary figures and images for: A gibberellin-assisted study of the transcriptional and hormonal changes occurring at floral transition in peach buds (Prunus persica L. Batsch)
Source: BMC Plant Biol. 2024 Jul 8;24:643. doi: 10.1186/s12870-024-05360-6 (PMC11229236; doi:10.1186/s12870-024-05360-6)

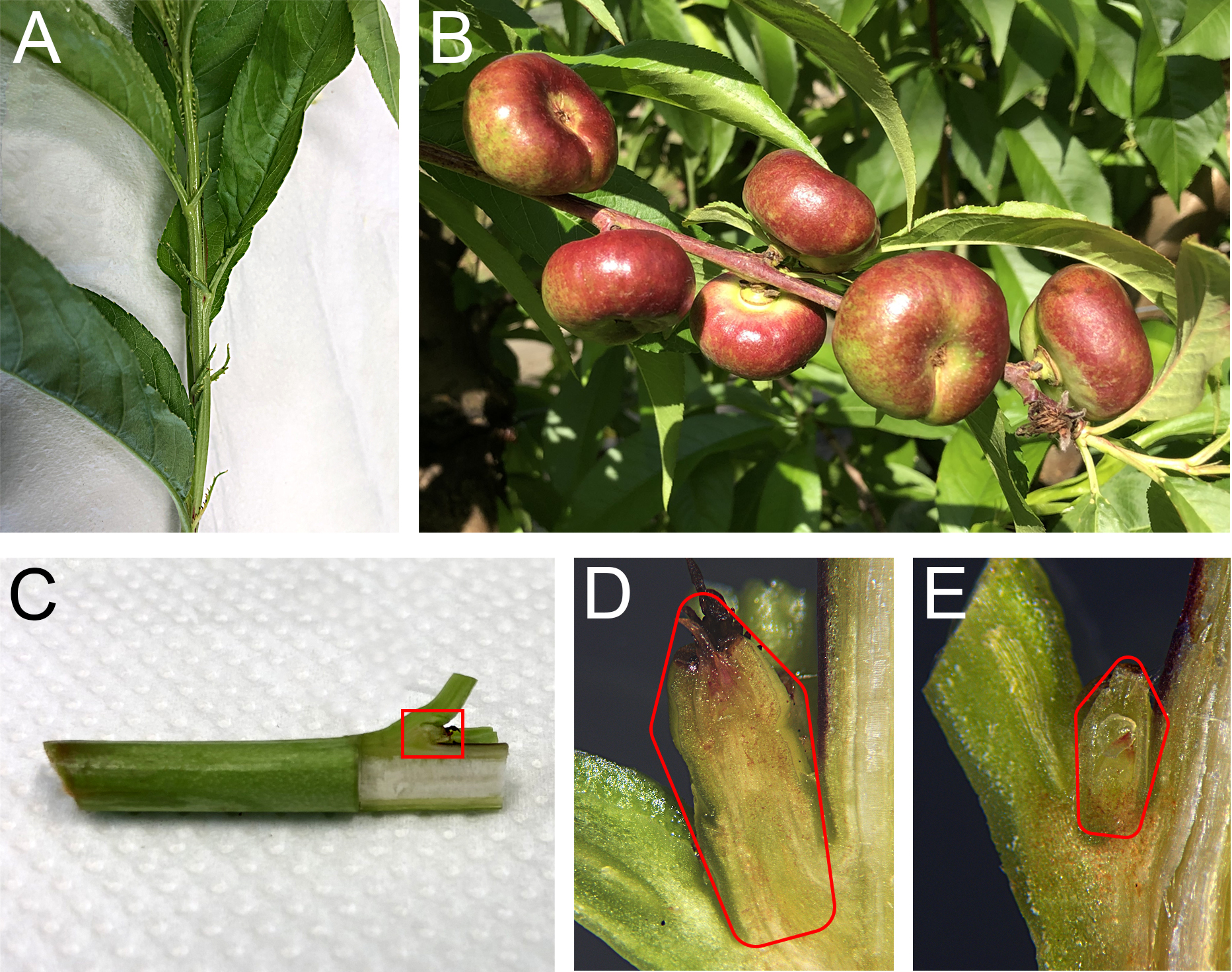

Supplement: Supplementary file 1 — Supplementary Material 1 [file 12870_2024_5360_MOESM1_ESM.jpg]

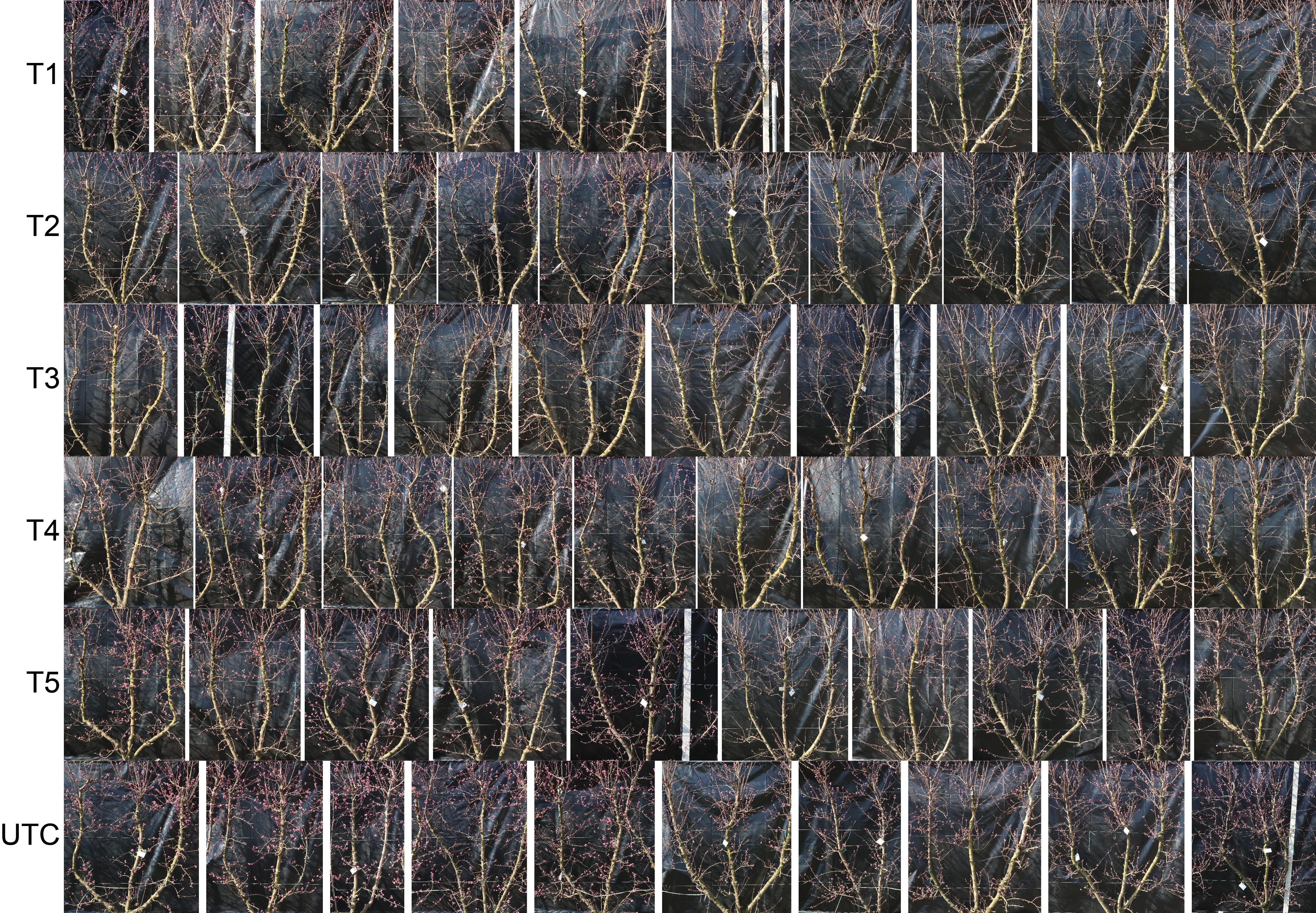

Supplement: Supplementary file 2 — Supplementary Material 2 [file 12870_2024_5360_MOESM2_ESM.jpg]

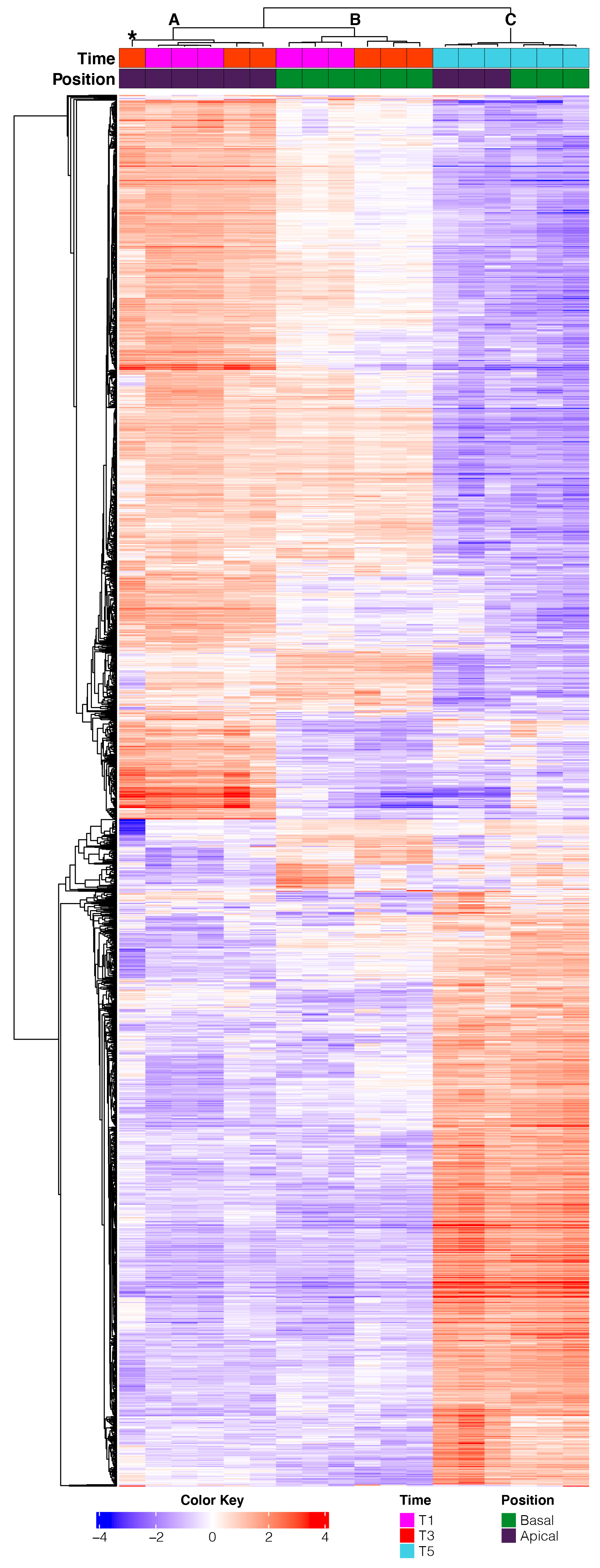

Supplement: Supplementary file 3 — Supplementary Material 3 [file 12870_2024_5360_MOESM3_ESM.jpg]

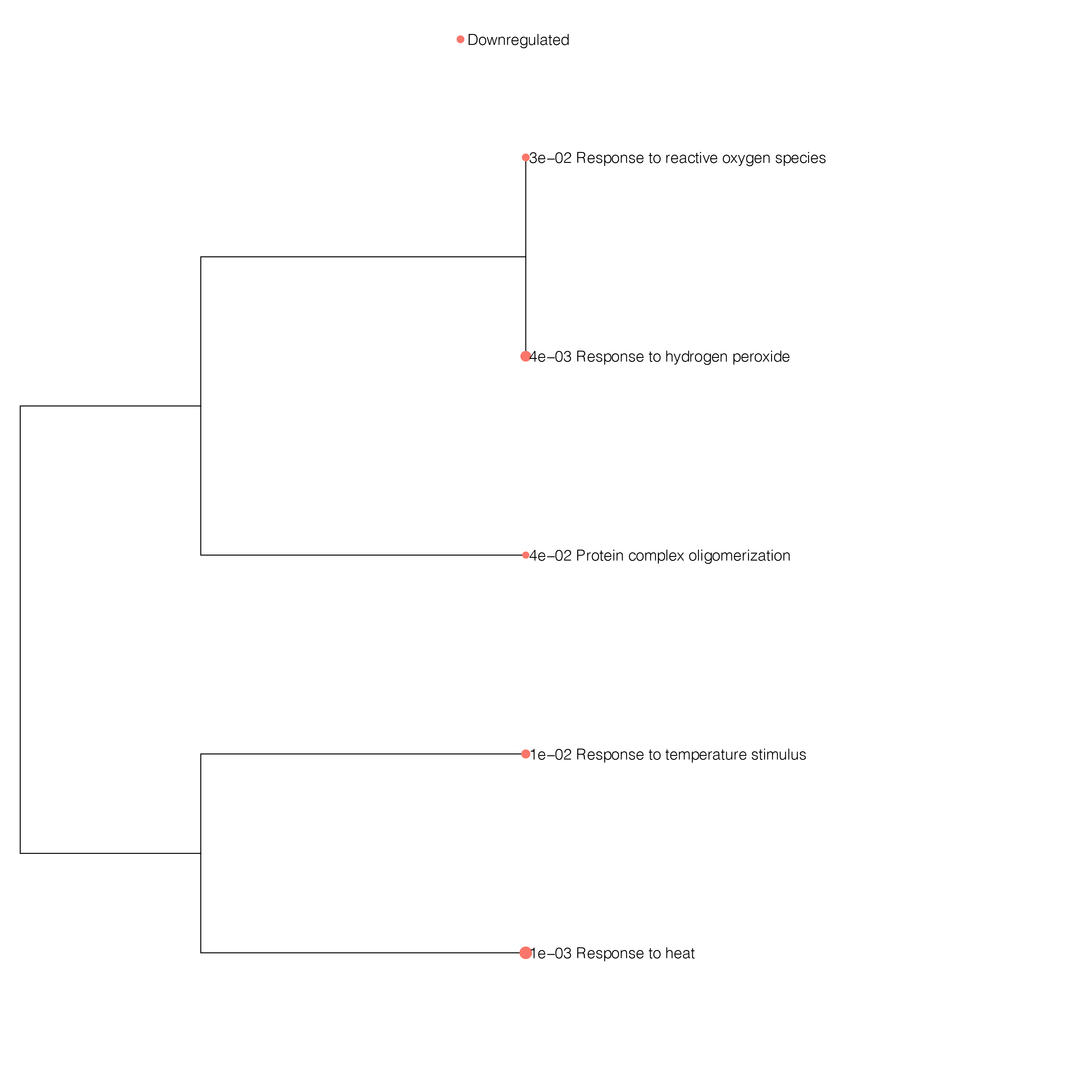

Supplement: Supplementary file 4 — Supplementary Material 4 [file 12870_2024_5360_MOESM4_ESM.jpg]

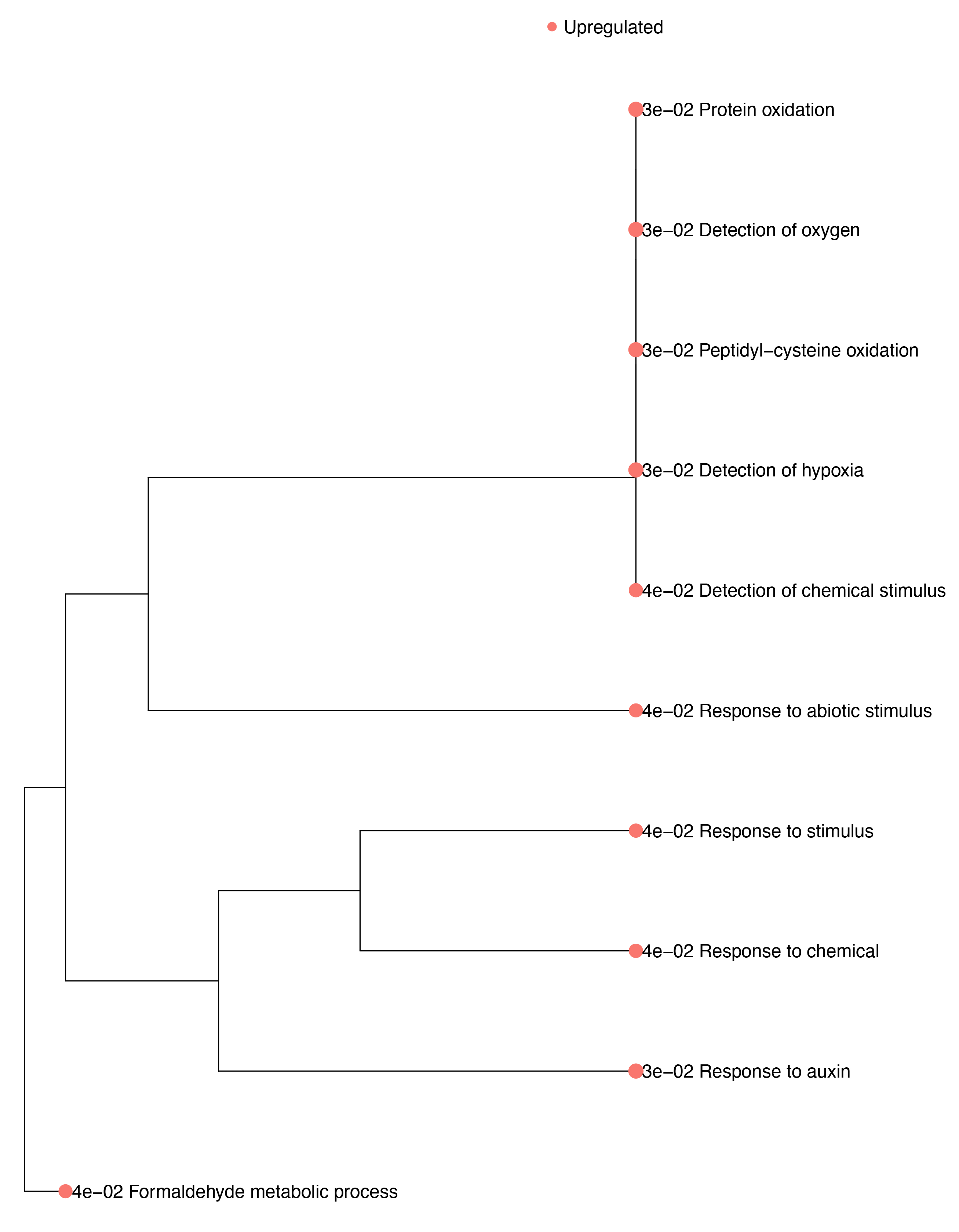

Supplement: Supplementary file 5 — Supplementary Material 5 [file 12870_2024_5360_MOESM5_ESM.jpg]

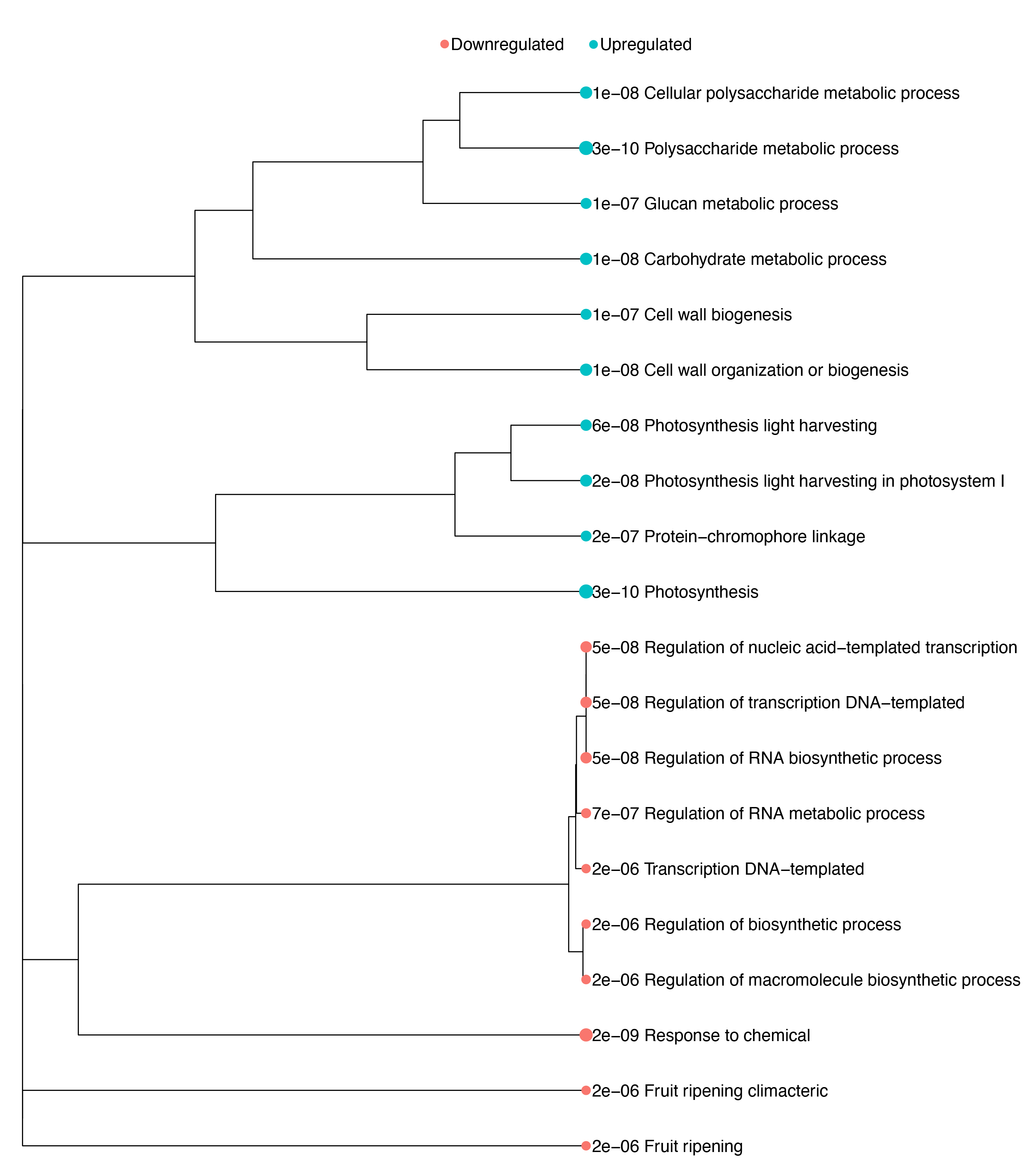

Supplement: Supplementary file 6 — Supplementary Material 6 [file 12870_2024_5360_MOESM6_ESM.jpg]

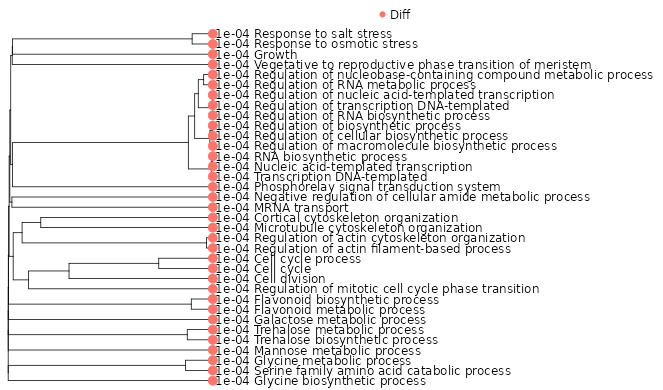

Supplement: Supplementary file 7 — Supplementary Material 7 [file 12870_2024_5360_MOESM7_ESM.jpg]

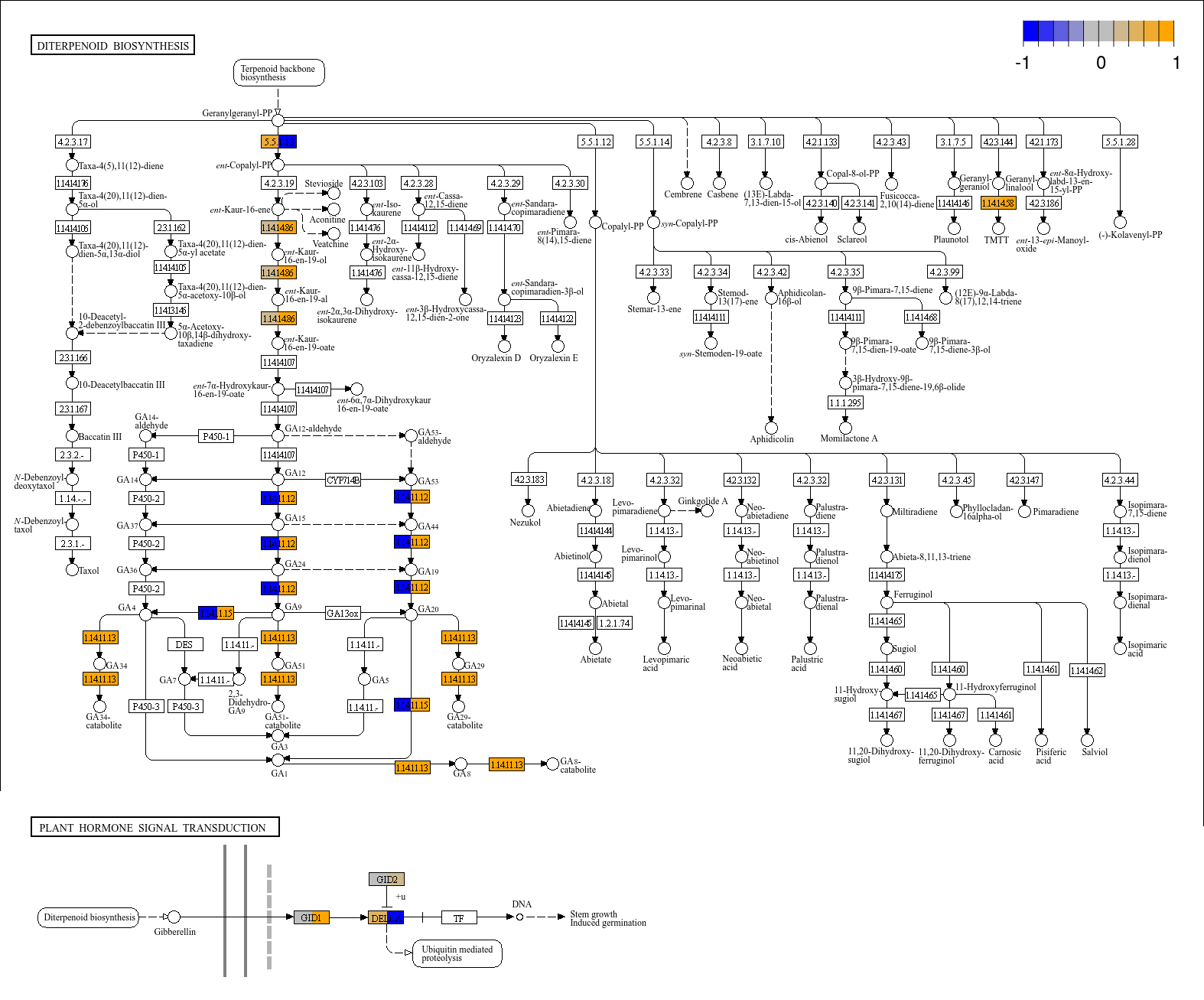

Supplement: Supplementary file 9 — Supplementary Material 9 [file 12870_2024_5360_MOESM9_ESM.jpg]

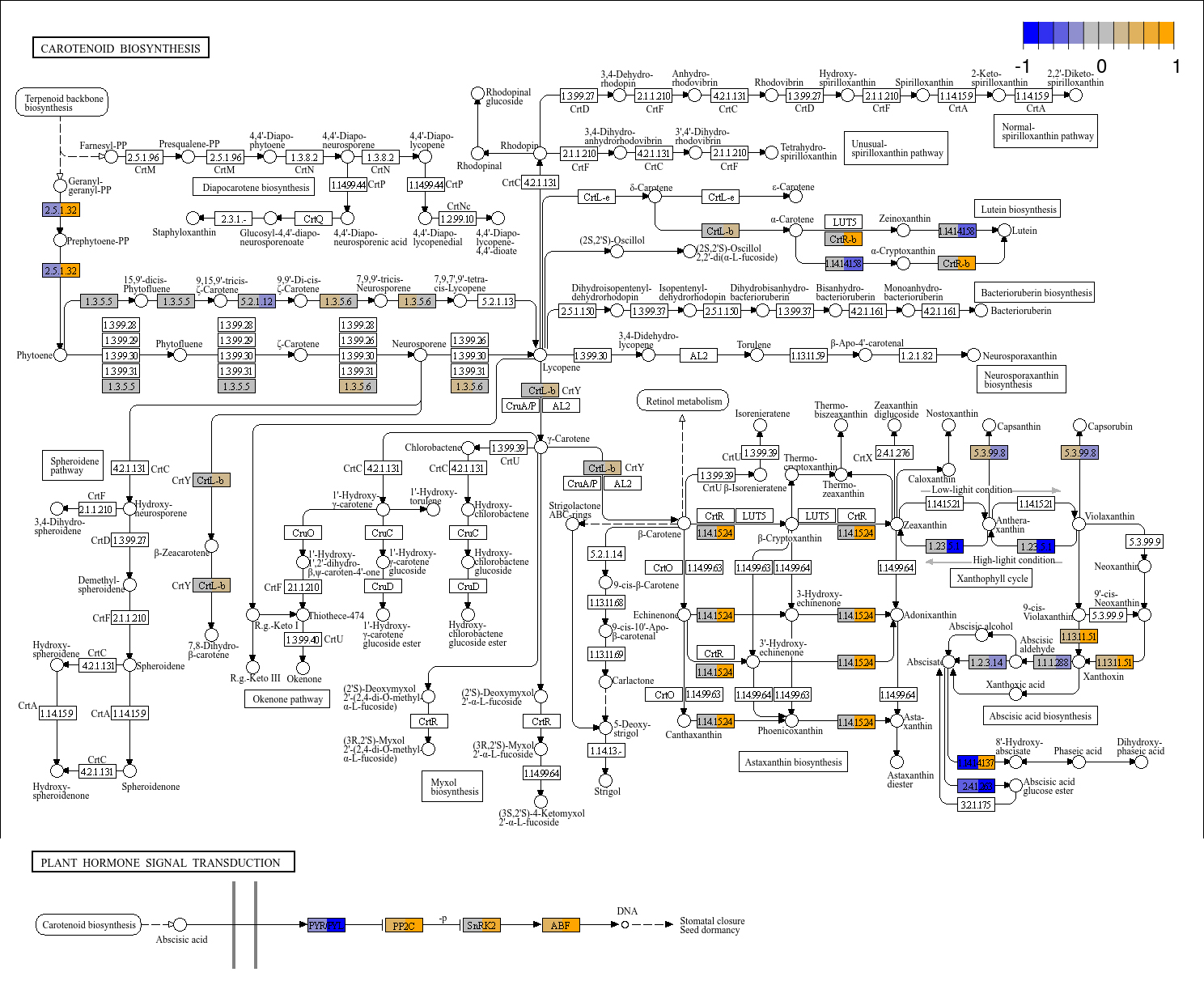

Supplement: Supplementary file 10 — Supplementary Material 10 [file 12870_2024_5360_MOESM10_ESM.jpg]

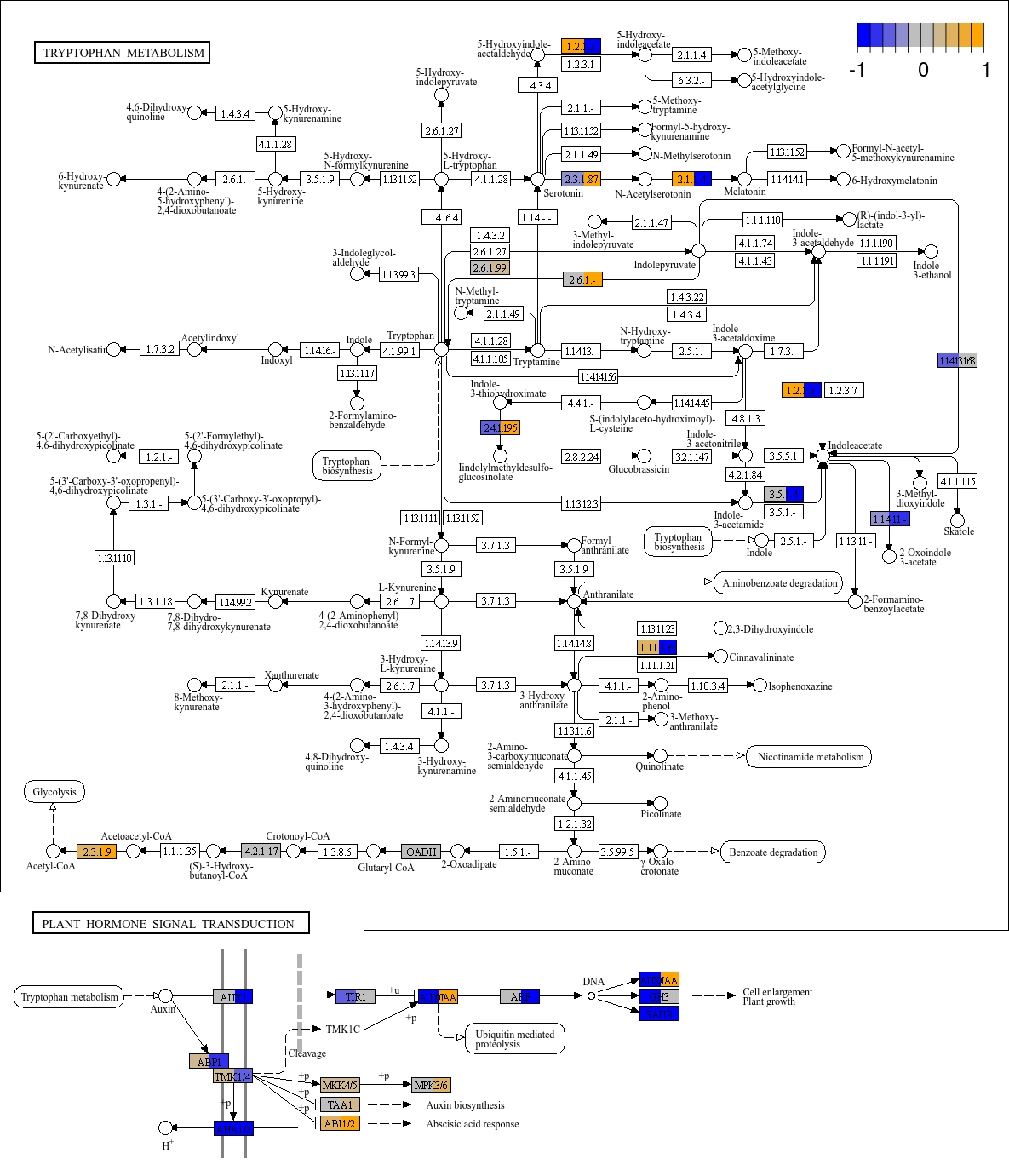

Supplement: Supplementary file 11 — Supplementary Material 11 [file 12870_2024_5360_MOESM11_ESM.jpg]

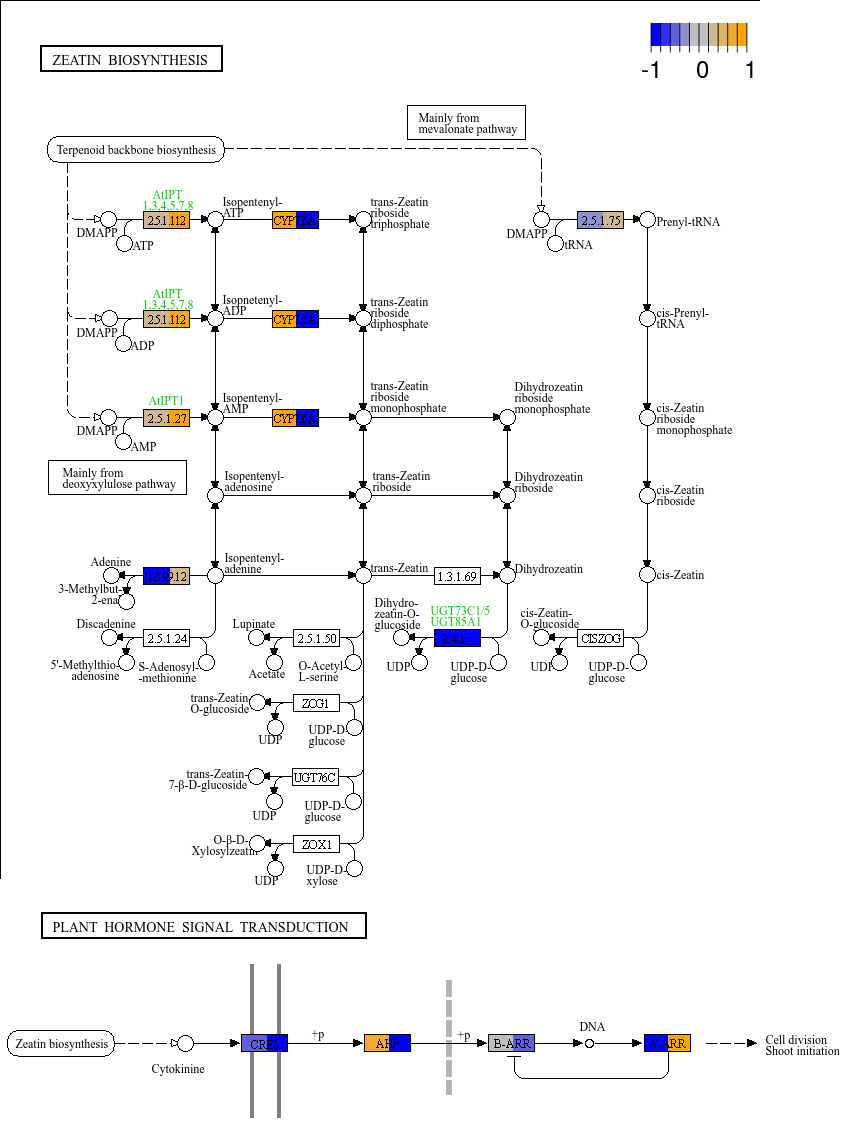

Supplement: Supplementary file 12 — Supplementary Material 12 [file 12870_2024_5360_MOESM12_ESM.jpg]

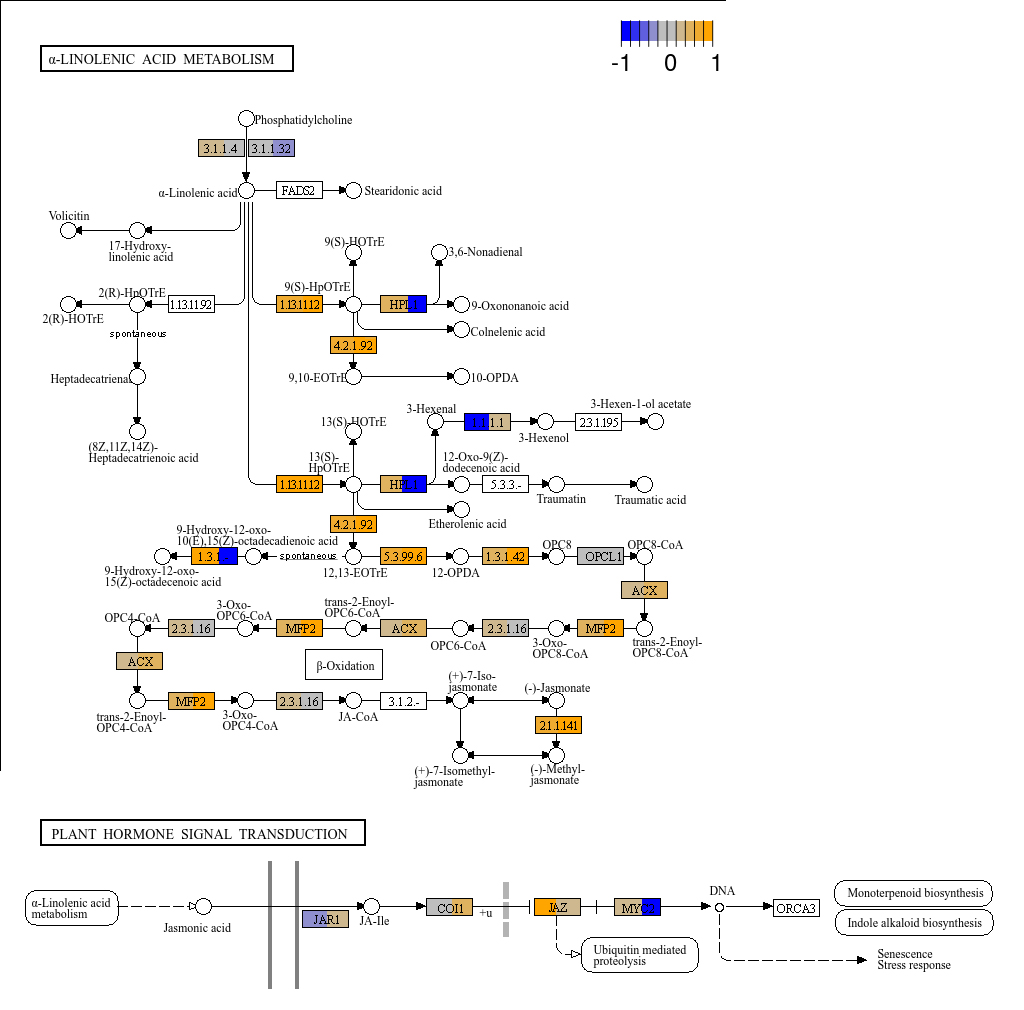

Supplement: Supplementary file 13 — Supplementary Material 13 [file 12870_2024_5360_MOESM13_ESM.jpg]

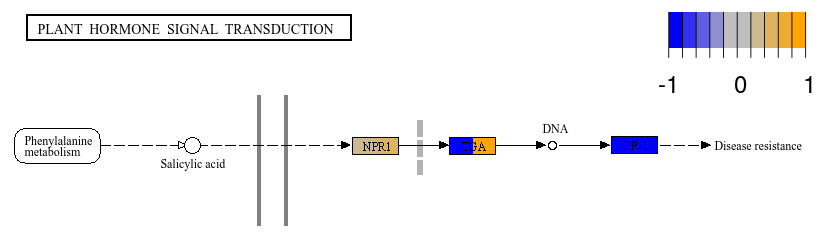

Supplement: Supplementary file 14 — Supplementary Material 14 [file 12870_2024_5360_MOESM14_ESM.jpg]

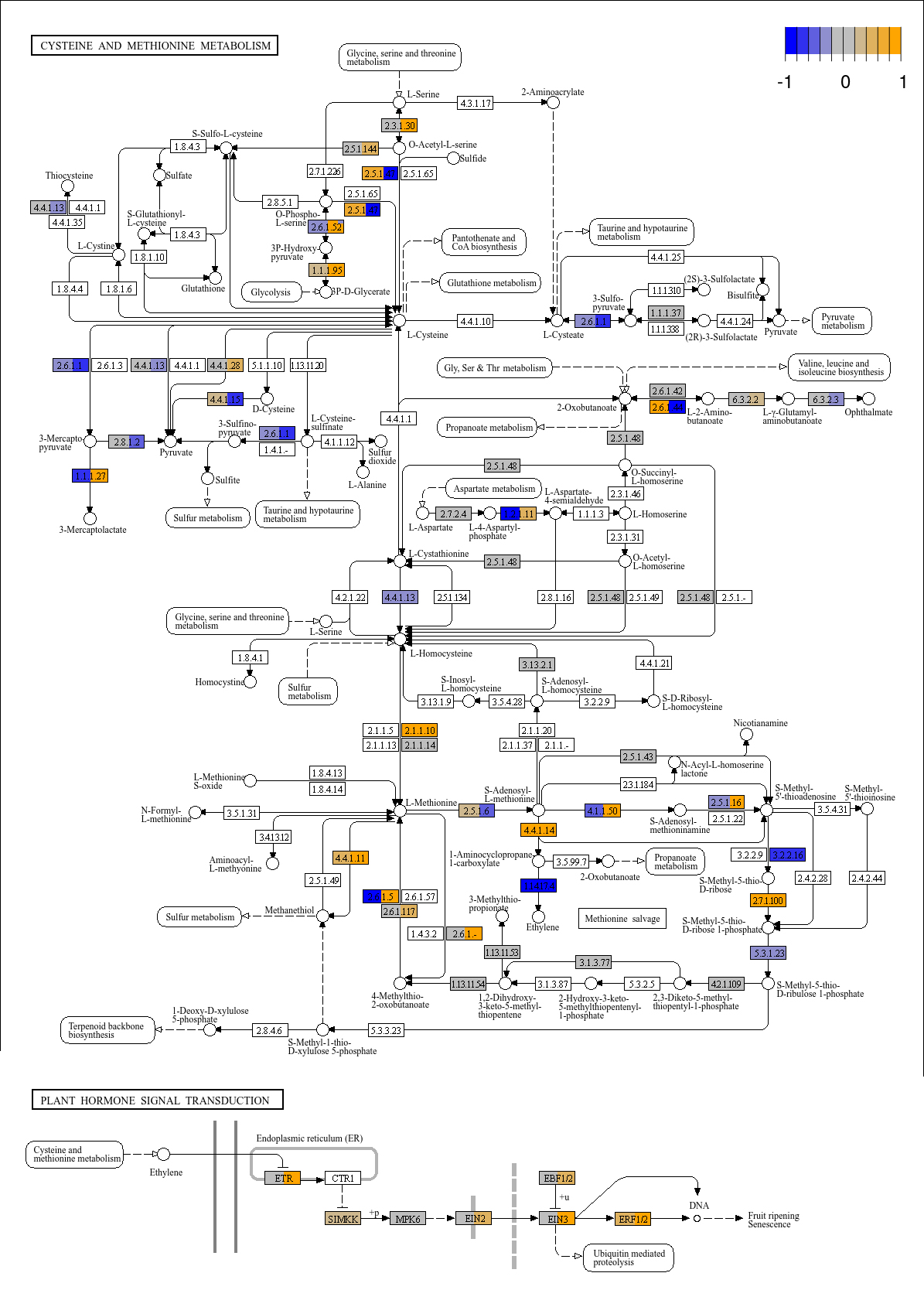

Supplement: Supplementary file 15 — Supplementary Material 15 [file 12870_2024_5360_MOESM15_ESM.jpg]
