# Supplementary material for: A gibberellin-assisted study of the transcriptional and hormonal changes occurring at floral transition in peach buds (Prunus persica L. Batsch)
Source: BMC Plant Biol. 2024 Jul 8;24:643. doi: 10.1186/s12870-024-05360-6 (PMC11229236; doi:10.1186/s12870-024-05360-6)
Supplement: Supplementary file 16 — Supplementary Material 16 [file 12870_2024_5360_MOESM16_ESM.docx]

# **Additional legends**

**Additional file 1 (.jpg)**

**Details about sample collection.**

One of the sampled peach shoots (A) and the fruit on the tree (B) at the beginning of the trial (45 DAFB). The localization of the sampled buds at the axil of the leaf (red box) (C) and sections of a basal (D) and apical (E) buds under a stereoscope at 65 DAFB. The red shapes mark the parts that were sampled at each timepoint.

**Additional file 2 (.jpg)**

**Trees at full bloom.**

Pictures of the trees of all treatments plus the UTC at full bloom in 2020. Only the cropped parts that were used for return bloom quantification with ImageJ are shown.

**Additional file 3 (.jpg)**

**Heatmap obtained with the 2,000 most variable genes in all samples and replicates.**

The timepoint and type of bud (*i.e*., position) are indicated at the top of the heatmap with different colors (legend in the bottom-right). Expression levels are reported with a color scale as shown in the bottom-left. The main clusters are named with capital letters, while the asterisk marks an outlier replicate.

**Additional file 4 (.jpg)**

**Enrichment analysis tree of the DEGs identified according to the efficacy of the treatment.**

The GO biological process terms have been used, along with the corresponding FDR statistics of significance.

**Additional file 5 (.jpg)**

**Enrichment analysis tree of the DEGs identified in the contrast T1 *vs* T3.**

The GO biological process terms have been used, along with the corresponding FDR statistics of significance.

**Additional file 6 (.jpg)**

**Enrichment analysis tree of the DEGs identified in the contrast T3 *vs* T5.**

The GO biological process terms have been used, along with the corresponding FDR statistics of significance.

**Additional file 7 (.jpg)**

**Annotation tree of PGSEA analysis.**

The hierarchy of the GO terms and the FDR statistics of significance resulting from PGSEA analysis are shown, which correspond to the pathways that are differentially expressed.

**Additional file 8 (.xlsx)**

**List of DEGs and enrichment analyses.**

Table S1: List of DEGs related to treatment efficacy, identified through DESeq2 with FDR < 0.01 and fold change > 2.

Table S2: List of DEGs related to bud development (i.e. the time of the treatment), identified through DESeq2 with FDR < 0.01 and fold change > 2.

Table S3.: GO-terms enriched in the genes that are correlated with 2-iP. A GO identifier, the P-value and corrected P-value of significance and a description of the GO-term are provided.

Table S4: GO-terms enriched in the genes that are correlated with JA-Ile. A GO identifier, the P-value and corrected P-value of significance and a description of the GO-term are provided.

Table S5: GO-terms enriched in the genes that are correlated with GA_7_. A GO identifier, the P-value and corrected P-value of significance and a description of the GO-term are provided.

Table S6: GO-terms enriched in the genes that are correlated with GA_4_. A GO identifier, the P-value and corrected P-value of significance and a description of the GO-term are provided.

Table S7: GO-terms enriched in the genes that are correlated with zeatin. A GO identifier, the P-value and corrected P-value of significance and a description of the GO-term are provided.

**Additional file 9 (.jpg)**

**KEGG pathway analysis of gibberellin biosynthesis and signal transduction.**

The diterpenoid biosynthesis pathway containing gibberellin biosynthesis (map No. 00904) and an extract of the signal transduction pathway of the same hormones (map No. 04075) are represented according to the standard KEGG visualization and symbols. The differential expression levels were calculated as log2-ratio, scale-centered for each time point, and color-coded as displayed in the legend (blue: <–1; orange: >+1). Each box, representing a regulatory step, is divided in two sub-boxes representing the two different contrasts (T3 *vs* T1 and T5 *vs* T3).

**Additional file 10 (.jpg)**

**KEGG pathway analysis of ABA biosynthesis and signal transduction.**

The carotenoid biosynthesis pathway containing ABA biosynthesis (map No. 00906) and an extract of the signal transduction pathway of the same hormone (map No. 04075) are represented according to the standard KEGG visualization and symbols. The differential expression levels were calculated as log2-ratio, scale-centered for each time point, and color-coded as displayed in the legend (blue: <–1; orange: >+1). Each box, representing a regulatory step, is divided in two sub-boxes representing the two different contrasts (T3 *vs* T1 and T5 *vs* T3).

**Additional file 11 (.jpg)**

**KEGG pathway analysis of IAA biosynthesis and signal transduction.**

The tryptophan metabolism pathway containing IAA biosynthesis (map No. 00380) and an extract of the signal transduction pathway of the same hormone (map No. 04075) are represented according to the standard KEGG visualization and symbols. The differential expression levels were calculated as log2-ratio, scale-centered for each time point, and color-coded as displayed in the legend (blue: <–1; orange: >+1). Each box, representing a regulatory step, is divided in two sub-boxes representing the two different contrasts (T3 *vs* T1 and T5 *vs* T3).

**Additional file 12 (.jpg)**

**KEGG pathway analysis of cytokinin biosynthesis and signal transduction.**

The zeatin biosynthesis pathway containing cytokinin biosynthesis (map No. 00908) and an extract of the signal transduction pathway of the same hormone (map No. 04075) are represented according to the standard KEGG visualization and symbols. The differential expression levels were calculated as log2-ratio, scale-centered for each time point, and color-coded as displayed in the legend (blue: <–1; orange: >+1). Each box, representing a regulatory step, is divided in two sub-boxes representing the two different contrasts (T3 *vs* T1 and T5 *vs* T3).

**Additional file 13 (.jpg)**

**KEGG pathway analysis of jasmonate biosynthesis and signal transduction.**

The linoleic acid metabolism pathway containing jasmonate biosynthesis (map No. 00591) and an extract of the signal transduction pathway of the same hormone (map No. 04075) are represented according to the standard KEGG visualization and symbols. The differential expression levels were calculated as log2-ratio, scale-centered for each time point, and color-coded as displayed in the legend (blue: <–1; orange: >+1). Each box, representing a regulatory step, is divided in two sub-boxes representing the two different contrasts (T3 *vs* T1 and T5 *vs* T3).

**Additional file 14 (.jpg)**

**KEGG pathway analysis of salicylate signal transduction.**

An extract of the signal transduction pathway of salicylate (map No. 04075) is represented according to the standard KEGG visualization and symbols. The differential expression levels were calculated as log2-ratio, scale-centered for each time point, and color-coded as displayed in the legend (blue: <–1; orange: >+1). Each box, representing a regulatory step, is divided in two sub-boxes representing the two different contrasts (T3 *vs* T1 and T5 *vs* T3).

**Additional file 15 (.jpg)**

**KEGG pathway analysis of ethylene biosynthesis and signal transduction.**

The cysteine and methionine metabolism pathway containing ethylene biosynthesis (map No. 00270) and an extract of the signal transduction pathway of the same hormone (map No. 04075) are represented according to the standard KEGG visualization and symbols. The differential expression levels were calculated as log2-ratio, scale-centered for each time point, and color-coded as displayed in the legend (blue: <–1; orange: >+1). Each box, representing a regulatory step, is divided in two sub-boxes representing the two different contrasts (T3 *vs* T1 and T5 *vs* T3).
